# A systematic review of the types, workload, and supervision mechanism of community health workers: lessons learned for Indonesia

**DOI:** 10.1186/s12875-024-02319-2

**Published:** 2024-03-11

**Authors:** Sofwatun Nida, Agatha Swasti Ayuning Tyas, Nidya Eka Putri, Ayudina Larasanti, Aldhea Ayu Widoyopi, Rima Sumayyah, Saetia Listiana, Aufia Espressivo

**Affiliations:** 1 Research and Policy Division, Center for Indonesia’s Strategic Development Initiatives (CISDI), Jakarta, Indonesia; 2 Primary Health Care Division, Center for Indonesia’s Strategic Development Initiatives (CISDI), Jakarta, Indonesia

**Keywords:** Community health worker, Health intervention, Community health services, Primary health care, Community health worker management

## Abstract

**Background:**

Community health workers (CHWs) have demonstrated capability to improve various health indicators, however, many programmes require support in meeting their objectives due to subpar performance and a high rate of CHW attrition. This systematic review investigated the types of CHWs, their workloads, and supervision practices that contribute to their performance in different countries.

**Methods:**

The search was carried out in November 2022 in Medline, Embase, and Neliti for studies published in Indonesian or English between 1986 and 2022 that reported public health services delivered by CHWs who live and serve the community where they live but are not considered health professionals. The findings were synthesised using a thematic analysis to assess key factors influencing the performance of CHWs.

**Results:**

Sixty eligible articles were included in this review. CHWs were responsible for more than two diseases (*n* = 35) and up to fifteen, with more than eighteen activities. Their roles covered the human life cycle, from preparation for pregnancy, care for newborns, health for children, adolescents, and productive age to elderly individuals. They were also involved in improving environmental health, community empowerment, and other social issues hindering access to health services. They carried out promotive, preventive, and curative interventions. The CHW-population ratio varied from eight to tens of thousands of people. Some CHWs did not have a clear supervision system. Challenges that were often faced by CHWs included inappropriate incentives, inadequate facilities, insufficient mentoring, and supervision, many roles, and a broad catchment area. Many studies revealed that CHWs felt overburdened and stressed. They needed help to balance their significant work and domestic tasks.

**Conclusions:**

Effective planning that considered the scope of work of CHWs in proportion to their responsibilities and the provision of necessary facilities were crucial factors in improving the performance of CHWs. Supportive supervision and peer-supervision methods are promising, however, any CHW supervision required a detailed protocol. This systematic review emphasised the opportunity for CHW management system improvement in Indonesia.

**Supplementary Information:**

The online version contains supplementary material available at 10.1186/s12875-024-02319-2.

## Background

The roots of Community Health Worker (CHW) programmes trace back to the 1930s in China, where the first CHWs, known as "Farmer Scholars," were trained. They laid the groundwork for the Barefoot Doctors, a movement that saw over a million trained individuals from the 1950s to the 1970s. During the 1960s and 1970s, smaller CHW initiatives began to emerge in various countries, especially in Latin America. These early CHW programmes served as prototypes and inspiration for larger-scale initiatives in many low-income countries during the 1980s. Despite initial enthusiasm, many CHW programmes faced challenges and failures in the 1980s and 1990s [1].

After decades, CHWs have gained significant recognition as a critical resource in achieving national and global health goals [2]. CHWs’ role in addressing the shortage of healthcare workers has become increasingly vital due to their effectiveness and practical deployment [3], making them not only an essential bridge for communities to access health services in many countries [4], but also an actor to enhance equitable access to primary healthcare (PHC) across diverse populations [5]. CHWs have demonstrated their ability to reach marginalised groups [6], resulting in positive impacts that include improvements in clinical disease indicators, screening rates, health behavioural change [7], and maternal and child health [8]. In many low- and middle-income nations, including Indonesia, CHWs deliver health services to the community and serve as the first point of contact for health-related issues as part of primary healthcare approaches [6].

In Indonesia, CHWs originated from the national women's Family Welfare Movement (PKK) in the 1970s, with trained volunteers called *kader* conducting health and nutrition activities. This evolved into the *Pos Pelayanan Terpadu* (*Posyandu*) in the mid-1980s, formally recognised by the Ministry of Health [9]. In response to the escalating health issues, the government established Posyandu and additional CHWs to address a broader range of concerns, including non-communicable diseases, elderly health, and adolescent health. This is deemed necessary as the existing Posyandu primarily focuses on maternal and child health services. However, the segmented nature of health services proven ineffective due to the cross-cutting nature of many health targets.

In 2022, the Ministry of Health initiated a comprehensive approach to integrate and revitalise primary health services, including the Posyandu strengthening. The transformed Posyandu aims to provide services across the entire life cycle, catering to pregnant women, the elderly, and others in an integrated manner. This approach is reinforced by regular and planned home visits conducted by CHW to ensure a holistic and cohesive healthcare strategy [10]. Yet, it is imperative for the government to glean insights from past experiences and proactively anticipate potential challenges that might impede the success of the programme. This is particularly crucial since initiatives involving CHWs often encounter obstacles, and the reliability of CHWs can be undermined by various factors.

Despite their valuable contributions, CHWs often face burnout due to overwhelming workloads [11]. This can lead to demotivation and decreased performance, ultimately impeding the achievement of programme objectives [12]. Numerous studies have highlighted that CHWs frequently receive additional assignments beyond their primary responsibilities [13]. Their small numbers are often tasked with serving disproportionately large populations, making it challenging to provide adequate care to all in need [14]. One crucial question that arises is determining the ideal ratio of CHWs to population or identifying the maximum population size that can be effectively handled by a single CHW. The circumstances under which specialist or generalist CHWs are more suitable to be deployed are also essential to explore. This is closely related to the multifaceted responsibility imposed on CHWs. To address these challenges, understanding the supervisory mechanisms implemented in various countries is key. Effective supervision plays a significant role in optimising the performance of CHWs and can help regulate their workload to become more manageable [15, 16]. By providing appropriate support and guidance, supervisors can empower CHWs to work more efficiently and sustainably [16, 17]**.**

We used a systematic review method to gain evidence of the type, workload, and supervision mechanism of CHWs from various studies conducted in various countries. The analysis of this study was tailored to the local context of Indonesia to inform policymakers in improving the existing CHW programme.

## Methods

### Search strategy and procedure

The systematic review search was performed on three databases on different dates: Medline and Embase (November 18th, 2022) and Neliti (November 21st, 2022). We included Neliti to obtain more articles from the Indonesian context. All studies identified are written in Indonesian or English, published in a peer-reviewed journal, and a primary research. Articles reported as a secondary study (opinion pieces, editorials, conference abstracts, letters, and advocacy materials were excluded. The articles included in this study were only those with Open Access. Multiple keywords and alternative terms were compiled as prompts to find relevant articles for this systematic review (see Table S1 for detailed search strategy). Five sets of search terms were used:

1. “community health worker community health worker or village health worker or community health aide or cadre or family planning personnel or *kader kesehatan* or *kader posyandu*”, AND
2. “supervision terms, or charge or monitoring or evaluation or coordination or superintendent or control or assessing or administrative or management or overseeing or direction or directive or governance or regulation or operation”, OR
3. “workload or prevent or screening or surveillance or detecting or counselling or educate or promote or task or employment or function or role or capacity or skill or communicate”, OR
4. “expertise or generalization or ability or specialization or arrangement or types of CHW or types of cadre”, AND
5. “performance or effectiveness or quality or improvement”.

### Eligibility criteria

The inclusion and exclusion criteria to obtain eligible articles were structured according to the standard population, intervention, comparison, and outcome (PICO) approach (see Table 1).

**Table 1:** Inclusion and exclusion criteria

**PICO**	**Inclusion Criteria**	**Exclusion Criteria**
Population	• CHW is a person who is not a health worker but contributes to community health services. • CHWs as part of the community. • CHWs provide services to the community where they live. • CHWs have or never received formal basic training (training certificates are recognised but not formal education programmes such as university certificates or courses). • Community level.	• Health workers (nurses, midwives, doctors, other paramedics). • Social CHWs. • Peer counsellors (peer).
Intervention	Intervention is part of primary health services carried out by the government entities (health centres or clinics) or non government entities	Study interventions are intended for specific populations, such as victims of natural disasters, refugees, or nomadic communities
Comparison	There were no group comparisons in this study.	
Outcome	The study explains the success or failure of the intervention (output, outcome, or impact) and mentions the factors that influence it.	

### Study screening

All database search results were uploaded to Covidence, a systematic review platform [18]. Then, articles that met the inclusion and exclusion criteria of the study based on keyword searches were exported. The screening process was carried out in three stages. First, articles were filtered to remove any duplicates. Then, two independent reviewers screened the titles and abstracts of the included studies against the PICOS criteria. After that, the full texts of the remaining articles were reviewed by two independent reviewers. In case disagreement arose between the reviewers, a third reviewer would assist in resolution.

**Fig. 1:**
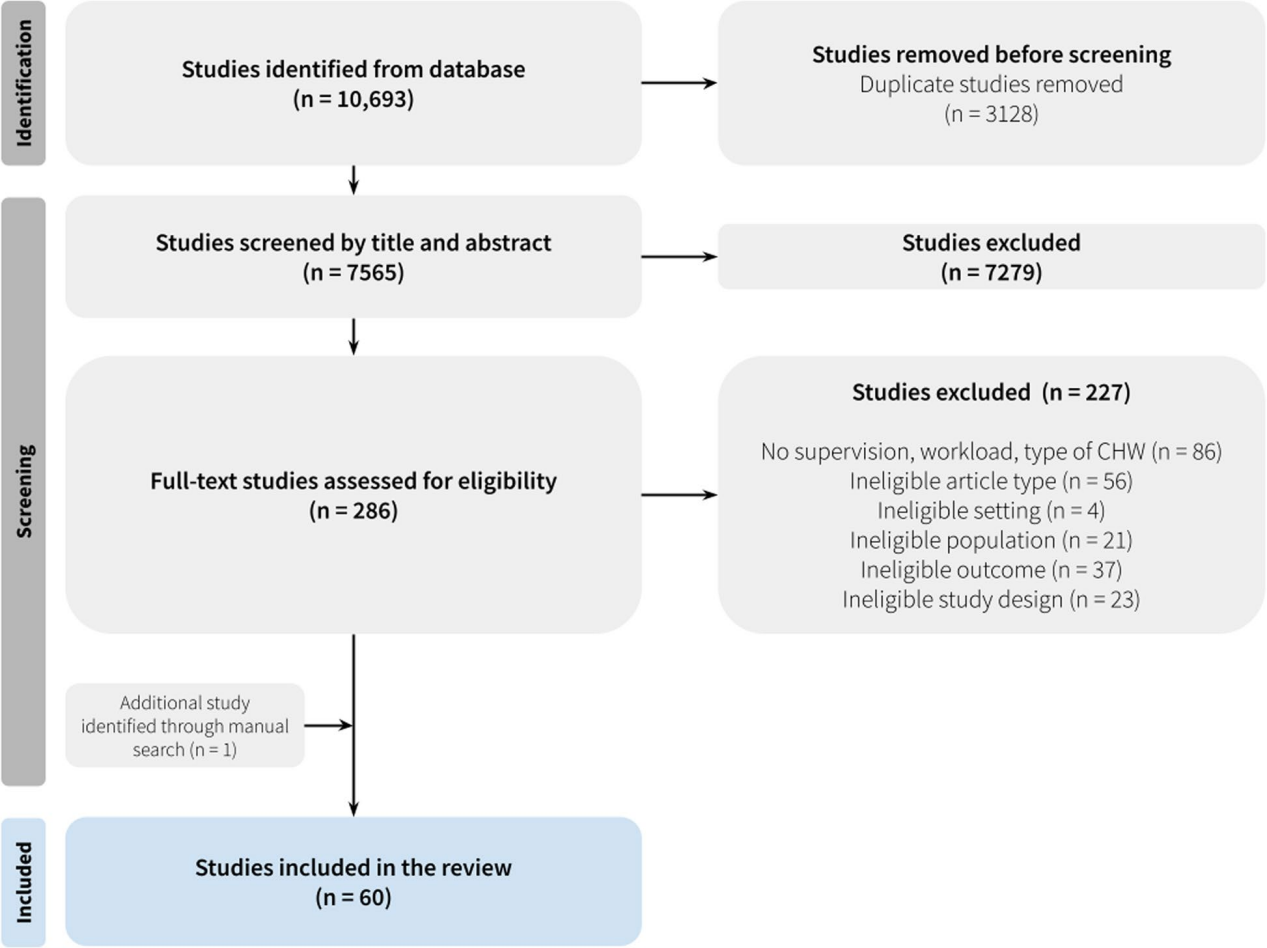
PRISMA Flow Diagram

### Data extraction

Following the screening, eight reviewers extracted ten categories of information from the article: title, author, year of publication, population, study location (country), health programme or intervention, CHW duties, supervision mechanisms, types of CHW, and intervention results (programme output or outcome). One independent reviewer checked the accuracy of at least 30% random sample from the extraction. The effect measures extracted were any type of outcomes that assess the efficiency or efficacy of the health programme, for example, health-related outcomes (e.g., mortality rate), service delivery outcomes (e.g., number of people being treated), and CHW-related outcomes (e.g., CHW depression or satisfaction).

### Quality appraisal

The quality of the selected studies was assessed by one reviewer and re-examined by another reviewer for at least 10% of the total studies using the Mixed Method Appraisal Tool (MMAT) version 2018 [19], a critical assessment tool designed for the assessment phase of systematic mixed study reviews. The MMAT consists of two screening questions and five criteria (questions), where each study for each criterion will be graded 1 (one) if it satisfies the requested criterion, 0 (zero) if it fails to satisfy the criteriona, and "not available" if it is not reported in the article. If the screening questions are marked 1 or "not available", it indicates that there is potential weakness in the study design and does not necessarily conclude that the research is not empirical. All eligible studies were included in the analysis.

### Data synthesis

Once all studies had been extracted using a standardised form, studies with similar outcomes were grouped into specific categories, such as CHW workload per population, and presented in a tabular format. Frequency calculations for studies within the same category were performed to identify trends on the workload and supervision of CHWs. The findings were synthesised using a thematic analysis. Despite the inclusion of various quantitative studies, a meta-analysis was not conducted due to the notable heterogeneity in methods and interventions across included articles.

## Results

### General descriptions

After duplicate articles were removed, we included 7,565 articles, of which; 286 articles were obtained in the title and abstract screening, manual search, and further assessment of full-texts yielded a total of 60 articles. A summary of the screening process is presented in the Preferred Reporting Items for Systematic Reviews and Meta-analyses (PRISMA) flow diagram (Fig. 1). Of the included articles, 53 discussed CHW workload and 50 examined CHW supervision mechanisms (see Table S3). Most studies were undertaken in East Africa (*n* = 25), South Asia (*n* = 12), and West Africa (*n* = 8), and the rest were in various regions, including South and Central Africa, Southeast Asia, the US, and Australia. In other words, most of the studies focused on low-middle-income countries (*n* = 51), with the remainder on upper-middle-income countries.

### Quality assessment

The articles quality assessment (see Table S2) shows that the majority of articles (*n* = 27) employed qualitative research methods, while others used non-randomised quantitative (*n* = 14), mixed methods (*n* = 14), and a randomised controlled trial (RCT) design (*n* = 5). The quality appraisals used the full sets of MMAT criteria (see Additional file); where a higher score indicates a better study quality. The results showed that, from 60 articles, 47 articles met 4–5 MMAT criteria, 7 articles met 3 criteria, and 6 articles met 1–2 criteria (see Table S2).

### Types of CHWs

Our findings showed that CHWs can be categorised based on the number of disease prevention programmes assigned to them or the population groups (see Table S3), namely, specialist and generalist CHWs, which is in line with WHO (2007) and Koon (2013) [20, 21]. A specialist CHW would provide a narrower range of services, responsible for no more than three disease prevention programmes that target the same population group. Meanwhile, generalist CHWs would be responsible for more than three programmes with multiple tasks and serve all age groups. We found only 53 articles reported CHWs’ workload, including 19 (34.5%) that highlighted specialist CHWs focusing on one to two diseases within the same population group and 36 (65.5%) discussing generalists CHWs responsible for managing various diseases across all age groups. Specialist CHWs typically addressed diseases necessitating specialised care and prompt treatment, including HIV, tuberculosis, malaria, diabetes mellitus, and paediatric conditions such as diarrhoea and pneumonia. They offered comprehensive health services, including curative treatments, with a narrower focus and specific outcome targets such as patient recovery and controlled blood pressure or blood sugar levels.

### The workload of CHWs

- Roles of CHWs

Most of the articles indicated that CHWs felt burdened by unrealistic workloads (*n* = 15) due to disproportionate population and task assignment, compounded by inappropriate incentives or benefits made available to them. Tasks assigned to CHWs, especially generalists, varied from country to country. In Brazil, one CHW could bear the responsibilities for 15 diseases and more than 18 programme activities, including pregnancy preparation and care for the newborns, health for children, adolescents, productive age, and elderly, all of which received community satisfaction with CHW performance [17]. In other countries, CHWs were also involved in efforts to improve environmental health [11, 17, 22–25], community empowerment, and other social issues [26–30] that hinder access to health services. These studies have made it evident that CHWs carry out promotive, preventive, and curative actions. Table 2 presents the roles of CHWs based on these three classifications.

**Table 2:** The roles of CHWs based on promotive, preventive, and curative classification

**Classification**	**Number of articles**	**Roles**
Promotive	42	Health promotion and education such as campaign and counselling (trusted sources of health information).
		Community mobilisation, empowerment.
Preventive	36	Health status monitoring, such as child growth and pregnant women.
		Provision of immunisation, contraception, vitamin A, and iron.
		Health screening, early detection (rapid test).
		Tracing, surveillance.
		Referrals.
		Supervision for patient treatment (monitoring medication adherence), follow up cases.
Curative	23	Provision of medical treatment (iCCM, malaria program).

*Abrreviations: iCCM,* integrated Community Case management

We found that most CHWs (*n* = 24) conducted their roles, such as health screening, monitoring child growth, health promotion, and health promotion, through monthly home visits. Kawasaki (2015) found that CHWs were expected by the community to assist in monitoring their health status through routine home visits, rather than provide strong medicine.

b) Catchment Area

The catchment area refers to the number of target populations assigned to each CHW. Table 3 shows that the catchment areas of CHWs were quite variable and distinguished by the number of patients, households, or population. Most papers identified that one CHW was responsible for 100–250 households (*n* = 11) [22, 26, 31–33] and fewer CHWs (*n* = 2) were in charge of more than 10,000 people (*n* = 3) [14, 34, 35]. CHWs who felt burnout were more likely overtasked in a catchment area with low CHW to population ratio [36]. However, those with higher ratios still experienced burnout despite having fewer tasks [14, 35]. CHWs handling 100 households showed many positive outcomes because their work was mostly supported by strong supervision, monthly remuneration, and adequate equipment [15, 17, 25, 27, 29, 35–41].

**Table 3:** Summary of the catchment area covered by the CHWs

**Catchment Area**	**Description**
< 50 patients (*n* = 7) [27, 36–39, 42, 43]	• CHWs had one or two responsibilities for disease prevention programme (specialist CHWs) [27, 36–39, 42, 43] • Most population had positive outcomes, namely, decreased child morbidity and mortality to improved blood glucose and weight control and increased access to acute malnutrition treatment in remote communities, reduced risk of repealed birth among teenagers 2 years) [27, 37–39, 42] • One study showed that high workload affected the CHWs duties and rendered distress, but did not specifically affect certain outcomes [36] • A study in Haiti suggested that the ideal ratio of CHW to patient is 1:4 with full-time work (40 hours per week) to address HIV and TB, and the ideal distance to walk from home to the patient was one hour round trip [43].
15—100 households (*n* = 9) [17, 26, 29, 30, 32, 40, 41, 44, 45]	• Most CHWs have more than three responsibilities for disease prevention programme (generalist CHWs) [17, 26, 30, 32, 40, 41, 44, 45] • Some households had positive outcomes, namely, community satisfaction of CHWs performance and improvement of antenatal care attendance [17, 29, 40, 41] • One study reported negatives outcomes, such as improved systolic blood pressure in hypertension, inconclusive effects on fasting blood glucose in diabetes, and no demonstrable effect on smoking (120 people per CHW, 40–60 working hours a month) [42, 44] • Some studies showed that public health programmes did not work optimally due to vague national policies and rushed implementation plans, and insufficient support for CHWs (transportation, remuneration, and supervision) [26, 32]
100—250 households (*n* = 11) [22, 31–33, 46–52]	• Most CHWs had more than two responsibilities for disease prevention programme (generalist CHWs) [22, 31–33, 46, 47, 50, 52] • All of them had a similar role to address mother and child health issues and communicable diseases [22, 31–33, 46–52] • Several articles mentioned that CHWs were overburdened due to excessive role, no training, weak supervision, and inappropriate incentive or compensation [22, 32, 47, 48, 51] • Those receiving positive outcomes facilitate CHWs with monthly salaries and essential support [33, 46, 49, 52]
More than 10.000 people (*n* = 3) [14, 34, 35]	• Two studies showed that CHWs experienced burnout and stress despite handling only one disease (malaria). It was attributed to the sizeable targets paired with inadequate and unsustainable support for training, compensation, supervision, access to equipment, and recognition [14, 34] • One study cited CHWs' success in 40,213 household visits, 127,011 health education sessions, and caring for 19,387 children under five. Their achievements were bolstered by in kind incentives like t-shirts, boots, umbrellas, solar power kits, training for coordinators, and three motorbikes [35].

c) Time commitment and remuneration of CHWs

We found that among 53 studies, 15 discussed CHW work durations and 13 addressed the provision of incentives. From 15 studies, CHW working hours spanned from 2 to 40 h per week, where three studies [32, 33, 53] reported that CHWs working for 2–4 hours received incentives [37], and one reported no incentives for CHWs. Aridi (2014) highlighted dissatisfaction among incentive recipients as they received incentives to compensate for additional tasks [32]. This emphasises the need for incentives based on working hours and targets. Gadsden’s research (2022) in Malang Regency, Indonesia, supported the idea that CHWs desired compensation proportional to their specific working hours [53]. Other studies indicated varying CHW working hours, ranging from 5–10 hours [48] to 10–20 hours per week [44, 54, 55], over 20 hours per week [46, 51] and even 40 or over weekly hours [14, 26, 43, 56]. In the latter case, some CHWs faced difficulties despite receiving incentives, with one study revealing that 14% of CHWs contemplated quitting.

Only one study by LeFevre analysed [48] the load and time commitment of CHWs, reporting that on average (median, 228 CHWs), each CHW only reached 25% of target households per year (around 120 home visits) despite earning monthly remuneration (USD 15.00). This means that one CHW reached only 10 households per month, while Ngugi (2018) mentioned 20 households per month [22].

### Supervision mechanism

There are four commonly discussed aspects or factors in the included studies that may impact the assessment of CHWs performance or productivity: supervisor position, supervision frequency, supervision method, and the number of supervisees.

- *Supervisor Position*

On the position of CHWs supervisor, many articles (*n* = 27) [11, 16, 17, 24, 26–28, 32–34, 42, 45–48, 50, 56–65] reported that CHWs supervision activities were mandated to health workers, all of whom worked at either the primary care or the lowest level of the health care facility. Seven articles [22, 29, 40, 44, 49, 55, 66] mentioned that supervision was conducted by a position that was working closely with CHWs, but they were not health workers or completing formal education. The nature of supervisors resembled that of a peer supervisor. Another type of supervisor was collaborative supervision by two or more people. Kenya had their CHWs supervised by the Community Health Committee (CHC) through monthly supervision to validate CHW reports before turning them further to the Community Health Extension Workers (CHEWs) [22, 32]. CHWs in Malawi worked in a household model, where CHWs were part of a three-tier structure locally called the Health Surveillance Assistants (HSAs), Senior CHW (SCHW), and Site Supervisor (SS). They worked together under the national CHW structure to assess clients’ identification and to coordinate activities, where each position had a clear job description [40] (see Table 4).

**Table 4:** Summary of CHW’s supervisor position

**Supervisor Position**	**Profession**	**Outcome**
Health Workers	The identified health workers were mostly professional nurses [26, 33, 48, 50, 56, 61], environmental health officers [11, 47], medical doctors [27], nurse or midwife, and others were unspecified.	Results varied. Some studies mentioned that supervision in the form of feedback and monitoring from healthcare workers resulted in positive output, such as increased perceived satisfaction of the community [17], good coordination during work [27], and increased motivation amongst CHWs [63]. Negative results caused by untrained supervisors [11], no standardised skills [65], unintegrated supervision conducted by several programme supervisors [11].
CHW (peer supervisor)	Senior CHWs, peer/colleague, CHEW (not health workers nor undertaking formal health education). In Pakistan, Lady Health Supervisor (LHS) observed and gave verbal feedback to Lady Health Workers (LHW) – a Pakistani term for CHWs. To become an LHS, one should have a minimum of eight years of primary education and previous experience as an LHW, and must reside within the area. In Uganda and Kenya, CHEWs were employed in the public health sector, and responsible for supervising CHWs [22, 67].	Generally, a positive outcome was related to CHW motivation. In Bangladesh, 68.4% of CHWs expressed satisfaction or motivation, while in Malawi it was 90.8% [31, 49]. Increase CHWs performance. Peer supervision was conducted in many countries like Malawi, India, and Uganda, with the increased CHWs performance was repeatedly mentioned to be the output of the programmes [29, 35, 65, 68].
Combination of supervisor	Community committees and CHEW [22, 32]; three-tiered structure [40].	Results depend on the clarity of supervisors’ scope of work and coordination pathway. In Malawi, the seemingly complex structure of mentorship resulted in CHW perceived positive responses and good health outcomes from end-beneficiaries to their performance. CHWs did not have any negative feedback on the supervision process [40].

b) *Supervision Frequency*

Seven articles [16, 17, 22, 31, 40, 69] mentioned supervision frequency, ranging from once a month to weekly sessions with varied results in CHWs’ performance or responses (see Table 5). While more frequent supervision sessions implied a more positive outcome, the method and quality of supervision played a large role in the successful performance of CHWs. Most government-led health programmes had supervision sessions more than once a month.

**Table 5:** Summary of supervision frequency and the outcome

**Example**	**Outcome**
Twice per month using peer feedback [31], site visit by supervisor [69]*,* weekly session from research staff [16].	Studies mentioned “effective in solving CHWs problems”, but supervisors mentioned that the biggest challenge was the expectation to cover a large geographical area.
Once per month using supportive supervision [17]	Improvement was reported in overall recognition and level of satisfaction regarding CHW performance among members of the community, from the baseline to the endline survey
Once per month [22]	CHWs who worked actively had less frequent supervision sessions than those who left the job or became inactive. CHWs who left the job reported having infrequent feedback from supervisors & overload work.
Once in three months [40, 70]	Ineffective supervision, leaving CHWs felt pressured to give reports to supervisors, saying “there is a gap between supervisor and CHWs.”

Positive outputs were noted in a health project in Brazil [17, 19] where CHWs were supervised by health professionals (unspecified types). The article mentioned that the supervision method created a partnership and collaboration process between health workers and CHWs. Some examples of negative output were evident in Kenya [22] which measured the incidence of CHW attrition and reported higher attrition in the group that received more frequent supervision because supervision “aimed at fault-finding rather than being supportive.”

c) *Supervision Method*

There were multiple methods of CHW supervision reported, both in the mechanisms and the instruments employed. Most health programmes used combined methods to  monitor CHW reports and their day-to-day activities or tasks. The former typically involved supervisors checking reports, registering quality assurance, or validating data (see Table 6).

**Table 6:** Supervision methods

**Method**	**Example**	**Findings**
Monitoring of CHWs reports	Report checking, registration of quality assurance, or data validation by supervisors.	The supervisor gathered CHWs once per month, and each CHW would present their report to the group [35]*.*
Observing CHWs day-to day activities	- Supervisors accompanied CHWs during household visits [17, 22, 29, 31, 47, 48] - CHWs were gathered in the village [34] - Remote supervision through digital communication or by phone [64] - CHWs made routine visits to health facilities to work under trained professionals [11]	- Supervisors and CHWs would discuss obstacles and opportunities of their work. - Site visits were also a method of leveraging CHWs capacities. This included face-to-face meetings to refresh the subjects given during pre-service training [17], or routine spot checks to assess CHWs performance to plan which would be used to plan future work [29, 48]. - CHWs in Bangladesh received a monthly check-in with the supervisors, through digital communication of at least once a month, and additional offline meetings if needed [64]. - A study using randomised controlled trials (RCT) in India reported that supervisors conducted the supervision activities remotely. An approximately five-minute call on a weekly basis discussed the feedback on CHWs target, work improvement, and technical problems faced by the CHWs. The reported outcomes included case activities and performance for duration in counselling by CHWs [38].
Supportive supervision		- Through this method, CHWs felt encouraged and valued [57, 71], and felt equal or at the same level as their supervisor [24]. - Supportive supervision was found to be meaningful during challenging and dynamic contexts, such as COVID-19 pandemic [64, 71]. - However, the supervision delivery method affected the supervision outcome. Trials in four countries showed a significant increase in CHWs motivation after supportive supervision through monthly group meetings [57]. - In a country where monthly group supervision was combined with biweekly peer supervision and self-assessment, CHWs showed significant improvement in their organisational commitment, work mindfulness, and confidence. - The expected downsides of this supportive supervision method is the complexity of preparing the supervisors to perform well. This included specialised training of mental health support [71], dedicated time allocation [24], and prequalification as graduate- or postgraduate-degree holder [66].

The supportive supervision method was mentioned several times in articles [24, 33, 36, 38, 40, 48, 49, 52, 57, 62, 64, 66, 71–73] with promising results. Supportive supervision is an accommodative and facilitative process where supervisors facilitate solutions for problems not only those related to medicine or public health, but also any social and psychological issues.

d) *Supervision Group Scale*

Many articles did not mention how many CHWs were supervised by one assigned supervisor. While fourteen articles mentioned the number of supervised CHWs, the range was enormous, from three people in a cluster RCT in India [44] to more than 50 people in a pilot project evaluation in Uganda [68].

Health programmes engaging larger groups (more than twenty CHWs per supervisory group) usually utilised peer-supervisory mechanisms, such as in India and Pakistan [31, 49, 66]. Meanwhile, health programmes in South Africa had professional nurses supervising approximately 21 CHWs [56], and Kenya combined health professionals and community health committees supervising approximately 25 CHWs [32].

## Discussion

### Types of CHWs

Our findings revealed a correlation between the CHW types, generalist and specialist, and the target population size as well as the programme workload. Ensuring the appropriate workload for CHWs may minimise potential burnout and hinder target achievement. Based on our systematic review, generalist CHWs were responsible for addressing various types of diseases and they could work effectively when they received a manageable workload, with an estimated coverage of under 100 households per CHW. Adequate support in the form of appropriate facilities such as fair remuneration, transportation, and necessary equipment was essential. These CHWs are best suited to concentrate on conducting home visits to oversee community health, which is in alignment with the preferences of the community [17].

In the Indonesian context, an integrated primary health service programme must be implemented [74] by engaging generalist CHWs to devise strategies to prevent the inefficiency of health services programmes for all age groups. When planning the tasks and scope of work of CHWs, relevant policymakers and decision-makers should consider lessons learned from other countries synthesised in this review. For example, inspiration can be drawn from Brazil's successful CHW programs that receive support in the form of regulations to ensure CHW welfare and accessible community facilities, thus contributing to CHWs’ achievements in improving public health outcomes. Brazil's experience offers valuable insights into Indonesian contexts that share common characteristics, such as a large population and vast geographical area [17].

### Identifying the optimum workload

Balancing the workload of CHWs is pivotal for programme success and community well-being. Workload should be customised according to their capabilities, available time (or working hours), and community accessibility. Addressing these concerns is vital to enhance CHWs’ effectiveness and job satisfaction.

We found that the ratio of CHW to population varied widely, with most areas having more than 100 households per CHW. The ideal ratio of CHWs to the population remains unclear, and, to our knowledge, no research with a robust method has addressed this issue. While some advocate for a 1:1000 ratio [75], others argue that this ratio, when applied to the population as a whole or to households, falls short of achieving desired outcomes [76]. Instead, more targeted ratios as potentially more effective alternatives are proposed, such as 1:600 population or 1:150 households [76]. The choice between these ratios depends on specific circumstances, resource availability, and the goals of the allocation.

We found one article that explained how programme makers determined the targets for CHWs [48]. It involves a calculation of the potential number of cases per year in a CHW work area and then distributed it over time to establish monthly or even daily targets. However, the evaluation results demonstrated that, on average, CHWs were only able to achieve 25% (120 households per CHW) of the set targets [48]. Therefore, determining CHWs' workload solely based on case estimates may not always be appropriate. This is especially true for programmes with voluntary participation schemes or limited incentives. Further research needs to focus on identifying relevant variables, particularly those related to the local context, to calculate the ideal workload for the CHWs.

### Peer vs supportive supervisions

Supervision mechanisms are highly contextual and dependent on various factors, including the clarity and division of roles between supervisors and CHWs, positive outcomes of supportive and peer supervisions, specific instruments, and the number of supervised CHWs in a single group. Implementing appropriate and well-thought-out supervision mechanisms based on the existing needs and available resources has proven to be effective in enhancing the quality of service delivery and performance of CHWs. On the other hand, evidence has shown that unclear, complex, and low-capacity supervision mechanisms can be frustating for CHWs [11, 47, 65].

Intuitively, more frequent and face-to-face sessions of supervision would have a more positive impact on CHW performance. However, the frequency of supervision alone is not the main factor in improving CHW performance. This review shows that the success of a supervision relies on the combination of both its frequency and methods. Studies also provide evidence of the success and good practices of peer and supportive supervisions.

Peer supervision can be a feasible and affordable option in areas with substantial catchment areas and a large number of CHWs. However, it is important to note that financial and time constraints can pose a challenge [68, 77, 78]. This is a promising method to adopt in Indonesia, where resources are limited. The recommended approach for peer supervision is to appoint high-performing CHWs as peer supervisors and provide them with additional training and support materials, for example, assessment tools on peer-supervisor selection and guidance for conducting supervision sessions. A set of predefined key performance indicators (KPIs) or checklists is a viable option for instruments as feedback references [29, 68]. The peer supervision mechanism provides a good opportunity for skill development between peers and improves the motivation of workers [79]. A similar approach has proven effective when applied to healthcare workers; nurses and midwives who gave routine peer review to each other had better service outcomes [80], and on-the-job peer training in vaccination for nurses had improved coverage [77].

Supportive supervision is a collaborative partnership where supervisors act as facilitators instead of inspectors. A facilitative or supportive supervision provides constructive feedback and facilitates supervisees in searching for solutions [24]. While several studies collectively recommended this method, it requires supporting factors. Complex methodology, in some articles, is the presumable aspect of supportive supervision that may hinder wide-range replications [57, 67]. The main characteristics of supervisors in the facilitative or supportive mechanism, according to Brown (2020), are being present to CHWs while providing safe space, constructive and constant monitoring, and coaching for CHWs.

Although repeatedly pictured as the most ideal form of supervision in the studies, supervision sessions needed some prerequisites prior to implementation. For example, supportive supervisors must have a certain depth of expertise and skills [81], commission additional external assistance (for the first initial sessions, training, and monitoring) [31], and dedicate abundant time allocation that also applies to CHWs. Therefore, supportive supervision should be considered in areas and conditions where prerequisite requirements are sufficient.

### Policy implication

The policy implications derived from our findings present a comprehensive framework for the advancement of CHW-based health programmes in Indonesia. First, the allocation of CHWs is not determined through workload calculations. For instance, in the case of maternal and child health CHWs (*kader posyandu*), the Ministry of Health determines the required number of CHWs based on five-stage services. Five CHWs are responsible for providing maternal and child services in a designated work area called a hamlet (RW or *dusun*) [82]. The number of households within an RW varies highly between districts; for example, the average household in one RW in North Jakarta (2019) is 116, while in Blitar City (2020), it is 60 [83]. Nonetheless, our findings suggest that this ratio remains balanced even when extending services to monitoring the health of all age groups (Posyandu PRIMA) through home visits, contingent on the government's resolute commitment to the programme. Essential to its success are comprehensive training for CHWs, clearly defined roles and working hours, fair compensation, adequate supervision, and supportive infrastructure. Our review provides learning that several programmes set ambitious targets without specifically addressing the corresponding commitment, leading to inadequate planning, funding, and essential resources for CHWs and supervisors. Vital resources such as transportation, appropriate remuneration, and clear programme guidelines are often lacking in most of our systematic reviews [11, 13, 14, 22, 24, 32, 34, 43, 45, 47, 48, 54, 58, 59, 61, 62, 64, 65, 71, 84].

Second, in Indonesia, CHWs receive substantial guidance from health workers at the Community Health Center (*Puskesmas*) in the form of relevant training to carry out their duties. However, the supervision to monitor CHW performance has not been fulfilled due to the overload duties of health workers. Therefore, we recommend that there should be regulations, prepared in the form of formal documents, to guarantee the availability of CHW supervisors and their supervision mechanisms. In the setting of organisation-based intervention, a protocol for operating procedure can be established [38], whereas in a larger sub-national or national context, a higher-functioned document or legal product such as Mayor Regulations or Ministry of Health and Ministry of Internal Affair Regulations [14, 85]. A formalised supervision mechanism is a good practice that contributes to a clear coordination of supervision sessions and positive outputs in our studies. These regulations may specify the personnel responsible for the supervision, the method, and the sought outputs or objectives.

Our findings underscore the importance of a well-defined workload distribution, effective supervision, and ample financial support for CHW operations. Consequently, the recommended policy action is to augment the budget allocated to the CHW programme and transfer budget authority to the Ministry of Health for increased flexibility in programme development. The current practice of CHW selection by village heads [86] or under the Ministry of Home Affairs presents several challenges in programme advancement, such as disparate CHW incentives, less robust supervision, and CHW turnovers with changes in the village leadership.

Strengthening the role of CHWs faces challenges without clear and detailed regulations delineating their rights and obligations. Implementation of minimum service standards by CHWs is crucial for enhancing service quality. Moreover, there is a need for the government to prepare CHWs as professionals rather than volunteers to amplify the impact of health service transformation.

### Strengths and limitation

The strengths of this systematic review lie in its comprehensive exploration of CHW workload, focusing on catchment areas and working hours. It also provides valuable insights into diverse supervision models used globally, an under researched area in systematic reviews. Furthermore, it aims to be applicable in the Indonesian context, serving as a practical resource for policymakers.

However, this study is limited by its reliance on a limited number of databases, potentially missing relevant research with diverse outcomes. The scarcity of published articles about CHWs in Indonesia limits a comprehensive understanding from the local perspective, despite efforts that have been made to include articles in Indonesia.

## Conclusions

The implementation of generalist CHWs may be suitable for Indonesia, especially alongside the rollout of the integrated primary health services. CHWs will be concentrated on health promotion and prevention of health concerns across all age groups. Despite the limited research on the optimal CHW-to-population or CHW-to-household ratio, successful examples from various nations demonstrate that CHWs overseeing fewer than 100 households showcase strong performance, even while managing diverse responsibilities. Clear guidelines from the central government are essential for CHWs, including qualifications, roles, workload, and reporting mechanisms. The recommended measures include establishing a supervision procedure to monitor and support CHW performance; regulating supervision methods to avoid overlapping roles; managing CHWs' workload; and accounting for population, accessibility challenges, as well as resources.

## Availability of data materials

Data is provided within the manuscript or supplementary information files (Table S3 Findings).

### Supplementary Information

**Supplementary Material 1.**

## Abbreviations

CHW: Community Health Worker
MMAT: Mixed Methods Appraisal Tools
PICO: Population, intervention, comparison, and outcome
PRISMA: The Preferred Reporting Items for Systematic Reviews and Meta-analyses
RCT: Randomised controlled trial
WHO: World Health Organization
HIV: Human immunodeficiency virus
iCCM: Integrated community case management
TB: Tuberculosis
CHC: Community health committee
CHEW: Community health extension worker
HSA: Health surveillance assistant
SCHW: Senior community health worker
SS: Site supervisor
LHS: Lady health supervisor
LHW: Lady health worker
KPI: Key performance indicator
