# Supplementary material for: A systematic review of the types, workload, and supervision mechanism of community health workers: lessons learned for Indonesia
Source: BMC Prim Care. 2024 Mar 11;25:82. doi: 10.1186/s12875-024-02319-2 (PMC10926673; doi:10.1186/s12875-024-02319-2)
Supplement: Supplementary file 1 — Supplementary Material 1. [file 12875_2024_2319_MOESM1_ESM.zip › Table S1 - Search strategy.docx]

Table S1 (a). Search strategy (Medline and Embase)

| **#** | **Query** | **Results from 18 Nov 2022** |
| --- | --- | --- |
| 1 | (community health worker or village health worker or community health aide or cadre or family planning personnel).mp. [mp=ti, ab, hw, tn, ot, dm, mf, dv, kf, fx, dq, bt, nm, ox, px, rx, ui, sy] | 14,700 |
| 2 | (supervision or charge or monitoring or evaluation or coordination or superintendent or control or assessing or administrative or management or overseeing or direction or directive or governance or regulation or operation).mp. [mp=ti, ab, hw, tn, ot, dm, mf, dv, kf, fx, dq, bt, nm, ox, px, rx, ui, sy] | 22,119,942 |
| 3 | (workload or prevent or screening or surveillance or detecting or counselling or educate or promote or task or employment or function or role or capacity or skill or communicate).mp. [mp=ti, ab, hw, tn, ot, dm, mf, dv, kf, fx, dq, bt, nm, ox, px, rx, ui, sy] | 18,308,015 |
| 4 | (expertise or generalization or ability or specialization or arrangement or types of CHW or types of cadre).mp. [mp=ti, ab, hw, tn, ot, dm, mf, dv, kf, fx, dq, bt, nm, ox, px, rx, ui, sy] | 2,679,428 |
| 5 | (performance or effectiveness or quality or improvement).mp. [mp=ti, ab, hw, tn, ot, dm, mf, dv, kf, fx, dq, bt, nm, ox, px, rx, ui, sy] | 9,128,096 |
| 6 | 2 or 3 or 4 or 5 | 37,278,142 |
| 7 | 1 and 6 | 10,647 |

**Database:**

Embase 1947-Present, updated daily

Ovid MEDLINE(R) and Epub Ahead of Print, In-Process, In-Data-Review & Other Non-Indexed Citations and Daily <1946 to November 17, 2022>

Table S1 (b). Search strategy (Neliti)

| **#** | **Query** | **Results from 18 Nov 2022** |
| --- | --- | --- |
| #1 | kader kesehatan | 26 |
| #2 | community health worker | 154 |
| #3 | kader posyandu | 28 |
|  | #1 OR #2 OR #3 | 58 |
