# Supplementary material for: A systematic review of the types, workload, and supervision mechanism of community health workers: lessons learned for Indonesia
Source: BMC Prim Care. 2024 Mar 11;25:82. doi: 10.1186/s12875-024-02319-2 (PMC10926673; doi:10.1186/s12875-024-02319-2)
Supplement: Supplementary file 1 — Supplementary Material 1. [file 12875_2024_2319_MOESM1_ESM.zip › Table S2 - MMAT Scores.docx]

**Table S2. MMAT Scores**

| **QUALITATIVE STUDY** | | | | | | | |
| --- | --- | --- | --- | --- | --- | --- | --- |
| **Study characteristics** | **Screening questions** | | **MMAT criterias** | | | | |
|  | Research question are clear | The collected data allow to address the research questions | Qualitative approach appropriate to answer the research question | Qualitative data collection method adequate to address the research question | The findings adequately derived from data | Results sufficiently substantiated by data. | Qualitative data sources, collection, analysis and interpretation are coherence |
| Rodriguez 2015 | 1 | 1 | 1 | 1 | 1 | 1 | 1 |
| Munshi 2019 | 1 | 1 | 1 | 1 | 1 | 1 | 1 |
| Hennein 2022 | 1 | 1 | 1 | 1 | 1 | 1 | 1 |
| Jerome 2010 | 1 | 1 | 1 | NA | 0 | 0 | 0 |
| Greenspan 2013 | 1 | 1 | 1 | 1 | 1 | 1 | 1 |
| Aridi 2014 | 1 | 1 | 1 | 1 | 1 | 1 | 1 |
| Smith 2014 | 1 | 1 | 1 | 1 | 1 | 1 | 1 |
| Kowitt 2015 | 1 | 1 | 1 | 1 | 1 | 1 | 1 |
| Datiko 2015 | 1 | 1 | 1 | 1 | 1 | 1 | 1 |
| Rabbani 2016 | 1 | 1 | 1 | 1 | 1 | NA | 1 |
| Ludwick 2018 | 1 | 1 | 1 | 1 | 1 | 1 | 1 |
| Assegaai 2019 | 1 | 1 | 1 | 1 | 1 | 1 | 1 |
| Karuga 2019 | 1 | 1 | 1 | 1 | 1 | 1 | 1 |
| Oluwole 2019 | 1 | 1 | 1 | 1 | 1 | 1 | 1 |
| Strodel 2019 | 1 | 1 | 1 | 0 | NA | NA | NA |
| Goudge 2020 | 1 | 1 | 1 | 1 | 1 | 0 | 0 |
| John 2020 | 1 | 1 | 1 | 1 | 1 | 1 | 1 |
| Ashebir 2021 | 1 | 1 | 1 | 1 | 1 | 1 | 1 |
| Ndambo 2022 | 1 | 1 | 1 | 1 | 1 | 1 | 1 |
| Musoke 2021 | 1 | 1 | 1 | 1 | 1 | 1 | 1 |
| Roy 2021 | 1 | 1 | 1 | NA | 0 | 0 | NA |
| Ishizumi 2021 | 1 | 1 | 1 | 1 | 1 | 1 | 1 |
| Pandya 2022 | 1 | 1 | 1 | 1 | 1 | 1 | 1 |
| Toney 2022 | 1 | 1 | 1 | 0 | 1 | 1 | 1 |
| Hill 2014 | 1 | 1 | 1 | 0 | 0 | 1 | 1 |
| Olaniran 2022 | 1 | 1 | 1 | 1 | 1 | 1 | 1 |
| Raven 2020 | 1 | 1 | 1 | 1 | 1 | 1 | 1 |

| **RANDOMISED CONTROLLED TRIALS (RCT)** | | | | | | | |
| --- | --- | --- | --- | --- | --- | --- | --- |
| **Study characteristics** | **Screening questions** | | **MMAT criterias** | | | | |
|  | Research question are clear | The collected data allow to address the research questions | Randomisation performed appropriately | The group comparable at baseline | Outcome data are complete | Outcome assessors blinded to the intervention provided | The participants adhere to the assigned intervention |
| Kaphle 2016 | 1 | NA | 1 | 1 | 0 | 1 | 1 |
| Aftab 2018 | 1 | 1 | 1 | 1 | 1 | 0 | 1 |
| Chin-Quee 2016 | 1 | 1 | 1 | 1 | 1 | 1 | 1 |
| Khetan 2018 | 1 | 1 | 1 | 1 | 0 | 0 | NA |
| Whidden 2018 | 1 | NA | 1 | 1 | 1 | 1 | 1 |

| **QUANTITATIVE NON RANDOMISED STUDIES** | | | | | | | |
| --- | --- | --- | --- | --- | --- | --- | --- |
| **Study characteristics** | **Screening questions** | | **MMAT criterias** | | | | |
|  | Research question are clear | The collected data allow to address the research questions | The participants are representative of the target population | The measurements are appropriate | Outcome data complete | The confounders accounted for in the design and analysis | The intervention administered as intended |
| Wroe 2021 | 1 | 1 | 1 | 1 | 1 | NA | 1 |
| Kok 2018 | 1 | 1 | NA | 1 | 1 | 1 | 1 |
| Shelley 2018 | 1 | 1 | 1 | 1 | 1 | 1 | 0 |
| Gadsden 2022 | 1 | 1 | 1 | 1 | 1 | NA | 1 |
| Kawakatsu 2022 | 1 | 1 | 1 | 1 | 1 | 1 | 1 |
| Ngugi 2018 | 1 | NA | 1 | 1 | 1 | 1 | 1 |
| VanBoetzelaer 2019 | 1 | 1 | 1 | 1 | 1 | 0 | 1 |
| Assegaai 2019 | 1 | 1 | 1 | 1 | 1 | NA | 1 |
| Bhattacharji 1986 | 1 | 1 | 1 | 1 | 1 | 0 | 1 |
| Kelly 2001 | 1 | 1 | 1 | 1 | 1 | 0 | 1 |
| LeFevre 2015 | 1 | NA | 1 | 1 | 1 | 1 | 1 |
| Kambarami 2016 | 1 | 1 | 1 | 1 | 1 | 1 | 1 |
| Dam 2022 | 1 | 1 | 1 | 1 | 1 | 0 | NA |
| Maravilla 2016 | 1 | 1 | 1 | 1 | 1 | 0 | NA |

| **MIXED METHODS STUDIES** | | | | | | | |
| --- | --- | --- | --- | --- | --- | --- | --- |
| **Study characteristics** | **Screening questions** | | **MMAT criterias** | | | | |
|  | Research question are clear | The collected data allow to address the research questions | Adequate rationale for using a mixed methods | The different components of the study effectively integrated | The outputs of the integration of qualitative and quantitative components | Divergences and inconsistencies between quantitative and qualitative results adequately addressed | The different components of the study adhere to the quality criteria of each tradition of the methods involved |
| Chin-Quee 2019 | 1 | 1 | 1 | 1 | 1 | 1 | 1 |
| Brenner 2011 | 1 | 1 | 1 | 1 | 1 | 1 | 1 |
| Collinsworth 2013 | 1 | 1 | 1 | 1 | 1 | 1 | 1 |
| Siekmans 2017 | 1 | 1 | 1 | 1 | 1 | 1 | 1 |
| Musoke 2019 | 1 | 1 | 1 | 1 | 1 | 1 | 1 |
| Kok 2019 | 1 | 1 | 1 | 0 | NA | 0 | 1 |
| Chipukuma 2020 | 1 | 1 | 1 | 1 | 1 | NA | 1 |
| Gottert 2021 | 1 | 1 | 1 | 1 | 0 | 0 | NA |
| Kawade 2021 | 1 | 1 | 1 | 1 | 1 | 1 | 1 |
| Nakibaala 2022 | 1 | 1 | 1 | 0 | 0 | 0 | 1 |
| Roy 2022 | 1 | 1 | 1 | 1 | 1 | 1 | 1 |
| O'Donovan 2022 | 1 | 1 | 1 | 1 | 1 | 1 | 1 |
| Revadi 2022 | 1 | 1 | 1 | 1 | 1 | 0 | 1 |
| Kawasaki 2015 | 1 | 1 | 1 | 1 | 1 | 0 | 1 |
