# Supplementary material for: A systematic review of the types, workload, and supervision mechanism of community health workers: lessons learned for Indonesia
Source: BMC Prim Care. 2024 Mar 11;25:82. doi: 10.1186/s12875-024-02319-2 (PMC10926673; doi:10.1186/s12875-024-02319-2)
Supplement: Supplementary file 1 — Supplementary Material 1. [file 12875_2024_2319_MOESM1_ESM.zip › Table S3 - Findings.docx]

Table S3 (a). Description of Community Health Workers Workload

| **Author Year**  **Location** | **Type and Name of CHWs** | **Health Issues** | **Population and working time** | **The Role of CHWs** | **Results/insight** |
| --- | --- | --- | --- | --- | --- |
| Kawasaki, 2015 [^20^](https://www.zotero.org/google-docs/?gAx6nS)  Brazil | Generalist, Community Health Workers  Receive monthly remuneration, were supported essential equipment | 1) Mother and child health (MCH; 2) sanitation and hygiene; 3) family planning; HIV/AIDS; 4) hypertension; 5) diabetes; 6) leprosy; 7) HIV/AIDS; 8) dermatitis and parasitic infection among children; 9) cervical cancer and breast cancer; 10) oral hygiene; 11) tuberculosis; 12) nutrition; 13) reproductive health; 14) health for older adults; 15) and people with psychophysical disabilities. | ~200 people or 1 community per CHW (40 HH) | 1) visiting each family in the catchment community at least once a month, 2) identification of risks and referral to the responsible sectors, 3) monthly monitoring of the growth of children under 2 years old (weight and height) and recording on a child monitoring card, 4) promotion of breastfeeding, 5) ensuring compliance with vaccination, 6) provision of guidance to families regarding oral rehydration salts to prevent diarrhoea and dehydration among children, 7) identification and registration of all pregnant women and accompaniment of them to antenatal care, 8) provision of guidance about family planning, 9) provision of guidance about HIV/AIDS prevention, 10) provision of guidance about prevention of infectious diseases, 11) monitoring of dermatitis and parasitic infection among children, 12) provision of health education about prevention of cervical cancer and breast cancer, 13) provision of health education about menopause, 14) provision of guidance on healthy food/nutrition, 15) provision of guidance on oral hygiene, 16) supervision of families with tuberculosis, leprosy, hypertension, diabetes, and other chronic disease patients, 17) provision of preventive care and promotion of health in the elderly, and 18) identification of people with psychophysical disabilities and provision of family support | **Regular home visit**: CHWs conducting home visits every month were well-received and appreciated by the community.  **Understanding of health conditions**: The community perceived that CHWs had a good understanding of the health conditions of family members. This indicates that CHWs were effectively assessing and addressing health concerns within households.  **Helpful home visit**: The community found CHWs' home visits to be helpful. This suggests that the interventions and advice provided by CHWs during their visits were valuable and beneficial.  **Overall performance satisfaction**: The community expressed overall satisfaction with CHWs' performance in maintaining the health of both individuals and families. This reflects positively on the effectiveness of CHWs' efforts in promoting community health. |
| Kaphle, 2016 [^39^](https://www.zotero.org/google-docs/?kjZsUD)  India | Specialist, Community Nutrition Expert | Neonatal health | Infant | 1) Visiting all the registered clients every two weeks; 2) submitting forms of infant health and nutrition and health status and; 3)tracking their nutritional progress at each follow-up visit; and 4) counselling families on infant nutrition in the last week for at least 15 minutes per family. | CHW's performance improved after receiving weekly supervision (supportive supervision) by phone call. |
| Rodriguez, 2015 [^8^](https://www.zotero.org/google-docs/?yUi1Qz)  Malawi | Generalist, Health Surveillance Assistants (HSA).  The HSAs are provided with bicycles, drug kits, have further educational opportunities and they receive a monthly salary of $100 | 1) Malaria, 2) pneumonia, 3) diarrhoea for children under five, 4) immunisations, 5) child growth monitoring, 6) water sanitation, 7) HIV, and 8) family planning | Not reported | 1) Conducting integrated community case management (iCCM) for childhood illnesses; 2) providing children under five immunisation services and child growth monitoring; 3) water sanitation; 4) disease surveillance; 5) health promotion; 6) supporting the health village committee; 7) providing family planning services, including injectable and oral contraceptive pills and condoms; 8) TB control activities; and 9) HIV testing and counselling. | HSAs are more likely to perform more tasks than those outlined in their job descriptions. Financial sustainability and health worker overburdening and Supervision were critical implementation issues that were identified during policy formulation but not resolved and have the potential to undermine the long-term prospects of iCCM.  Coverage of iCCM varied by district between 40.8 and 100%, national target 76%. |
| Chin-Quee 2019  [^10^](https://www.zotero.org/google-docs/?HpNOvv)  Burkina Faso | Generalist, Community Health Workers | 1) Mother and child health, 2) Family Planning | Not reported | In addition to carrying out their main duties, CHWs were also asked to provide family planning services, consisting of: 1) providing counselling about the complications of contraceptive use; and 2) carrying out injectable contraceptive services. | CHW felt burdened and received inappropriate incentives. |
| Aftab, 2018 [^30^](https://www.zotero.org/google-docs/?WWHpfD)  Pakistan | Specialist, Lady Health Workers (LHWs) | Childhood diarrhoea and pneumonia | 150-200 households per CHW | Conducting iCCM for childhood diarrhoea and pneumonia. | Supervisory performance of intervention arm supervisors was better than that in the comparison arm in correcting the workers clinical examination skill and more frequent feedback. In the household survey, only 18% intervention and 23% comparison arm caregivers considered LHWs capable of providing diarrhoea and pneumonia care. Commodities for integrated community case management were not regularly available to workers. |
| Ngugi, 2018 [^21^](https://www.zotero.org/google-docs/?PTAiss)  Kenya | Generalist, Community Health Workers | 1) Maternal, neonatal, and child health, 2) communicable disease (TB), 3) HIV, and 4) sanitation. | 150 households per CHW | 1) Encouraging people who were sick to seek medical attention; 2) asking pregnant mothers to deliver at health facilities; 3) giving lectures about exclusive breastfeeding and childhood immunizations; 4) Identifying tuberculosis (TB) and antiretroviral (ARV) medication defaulters; 5) encouraging the boiling or treatment of (drinking) water; 6) registering members of assigned households; and 7) conducting monthly household visits to identify health issues (and more regularly if there was a sick child or newborn). | CHWs mentioned working long hours to reach their target households. Initially, they would visit only 20 houses per month. However, when the government revised this to 100 households per month, targets became unachievable. Lack of a stipend or salary meant that they had to work extra hard to provide for their families. In some instances, volunteering took a lot of additional (unpaid) time and they often worked throughout the day without lunch. Sometimes the households visited would be empty at the time, so they had to make several re-visits, often over a number of days. There was also negative community feedback due to unrealistic expectations, for example, CHWs were expected to provide treatment for minor ailments such as coughs and jigger infestations.  Suggested improvements for CHW work environment: provide transport (motorcycles), offer ambulance services, give monetary incentives to alleviate family support concerns, reduce households covered to 20, and provide tokens like bags and *Khangas* (traditional printed linen), t-shirts and identification badges.  Absence of refresher training and receiving no feedback from supervisors were also associated with attrition. Discordance in expectations and perceived heavy workload were also identified as key reasons for attrition in the qualitative study. |
| Assegaai 2019 [^56^](https://www.zotero.org/google-docs/?QAMe4X)  South Africa | Generalist, Community Health Workers | 1) HIV/TB, 2) maternal and child health, 3) chronic disease | Not reported  Working time: everyday | Addressing HIV/TB, maternal and child health, and chronic disease care. | This study identified weaknesses in both the design and implementation of the supervision system of WBOTs. The lack of explicit, coherent and holistic guidance in policy and the failure to address constraints to supervision at local level undermine the performance and sustainability of the WBOT strategy in South Africa. |
| Munshi 2019 [^25^](https://www.zotero.org/google-docs/?Sjyt8j)  South Africa | Generalist, Ward-based Primary Healthcare Outreach Teams (WBPHCOTs) | 1) MCH, 2) non-communicable disease, 3) communicable disease (TB); 4) HIV and 5) social issues | 250 households per team (7-12 WBPHCOTs)  Working time: everyday | Carry out promotive and preventive care, including maternal and child health, non-communicable diseases, HIV and TB, an expansion of their previous roles in non-governmental organisations as mainly TB or HIV care workers. Furthermore, they had to operate as part of an integrated service with sectors such as social development, home affairs and education to address barriers to healthcare access | There were significant weaknesses in early implementation resulting from a vague national policy and a rushed implementation plan. During the installation stage, adaptations were made to address gaps including the appointment of sub district managers and enrolled nurses as team leaders.  Staff preparation of CHWs and team leaders to perform their roles was inadequate. To compensate, issues, such as CHWs receiving a stipend rather than being employed, were ongoing implementation challenges. Another challenge was that facility managers were employed by the local government authority while the CHW programme was perceived to be a provincial programme. |
| Hennein, 2022 [^14^](https://www.zotero.org/google-docs/?iAbAps)  Uganda | Specialist, Community Health Workers | Tuberculosis | Not reported | Provided education on TB, tracing, and screening the suspects. | CHWs identified five CoP activities as core to improving the quality of their work:  1) individual review of feedback reports,  2) collaborative improvement meetings,  3) real-time communications among members, 4) didactic education sessions, and  5) clinic-wide staff meetings.  CHWs reported that these activities provided a venue for them to share challenges, exchange knowledge, engage in group problem solving, and benefit from social support. CHWs also explained that they felt a shared sense of ownership of the CoP, which motivated them to propose and carry out innovations. CHWs described that the CoP strengthened their social and professional identities within and outside the group, and improved their self- efficacy. |
| Bhattacharji, 1986 [^46^](https://www.zotero.org/google-docs/?sWL5ny)  India | Generalist, Part-Time Community Health Workers (PTCHWs) | 1) Maternal and Child Health, 2) TB | 1000-1500 people per CHW  Working time: Part time | 1) Provided health education and encouraged pregnant women to seek health care; and 2) Monitored pregnant women, children under five, and TB patients through home visits. | The PTCHWs with the highest performance scores had, on the whole, less education, were more experienced, had a smaller population to cover, and received more intense supervision. |
| Kelly, 2001 [^59^](https://www.zotero.org/google-docs/?FDb42e)  Kenya | Generalist, Community Health Workers | 1) Child health, 2) family planning, and; 3) HIV | Not reported | Provided care for children with acute respiratory infections, diarrhoea, and malaria; referred severely ill children to health facilities. In addition, CHWs counsel caregivers on the continued care of their sick children at home and on behaviours related to health and disease prevention (e.g., immunizations, family planning, and preventing HIV infection) | CHWs often made mistakes assessing symptoms, classifying illnesses, and prescribing correct doses of medications. Key reasons for the deficiencies appear to be guideline complexity and inadequate clinical supervision. |
| Jerome, 2010 [^43^](https://www.zotero.org/google-docs/?8IWJDx)  Haiti | Specialist, Community Health Workers | 1) TB and; 2) HIV | 4-8 patients per CHW  Full time, 40 hours a week | 1) Preparing TB and HIV treatment, follow up, and observe treatment outcomes, identifying the side effects of therapy; and 2) Active case finding on HIV, TB and sexually transmitted infection (STI) in the population. | According to CHW, the ideal patient-CHW ratio is 4:1.  The ideal distance to walk from home to the patient is one hour round trip.  Even though they work full time, some of them were paid part time.  CHWs are a trusted source of health and non-health information for the community. |
| Brenner, 2011 [^37^](https://www.zotero.org/google-docs/?vMnx0s)  Uganda | Specialist, Volunteer Community Health Workers | Child health | 45 children under age five years each, living in 25 households. | (1) Offered health information to families with children under five through home visits and health talks; (2)encouraged families to attend health outreach activities including National Child Health Days; (3) supported families with ill children and counselled on appropriate home treatments; (4) referred children and pregnant women showing danger signs to nearest health centre; (5) followed up children who were recovering from illness; (6) supported children with special needs; (7) conducted monthly report; and (8) community development. | A low-cost child health promotion model using volunteer community health workers demonstrated decreased child morbidity, dramatic mortality trend declines and high volunteer retention. |
| Greenspan, 2013 [^22^](https://www.zotero.org/google-docs/?URYe1X)  Tanzania | Generalist, Community Health Workers | 1) Sanitation, 2) nutrition, 3) maternal, neonatal, and child health, and 4) HIV | Not reported | 1) Environmental sanitation campaigns; 2) distributing medicines and nutritional supplements; 3) conducting home visits to pregnant women and children; 4) assisting nurses during outreach campaigns; 5) monitoring children's growth and development; 6) reminding HIV-positive patients to take medication; 7) providing referrals to primary health care; 8) distributing condoms; 9) responding to emergencies; and 10) providing health education. | Sources of CHW motivation were identified at the individual, family, community, and organisational levels. Supervision can be dis-incentivizing if perceived as a sign of poor performance |
| Collinsworth, 2013 [^26^](https://www.zotero.org/google-docs/?qkPT9v)  USA | Specialist, Community Health Workers | Diabetes mellitus | Maximum 6 patients per CHW over 12 months. | 1) Educating uninsured and underserved patients on: general knowledge of diabetes, social support, diet, physical activity, and access to care; 2) Clinical assessments per quarter on: A1C, blood pressure, weight, and foot condition (visual and monofilament assessment); 3) promote self-management behaviours and facilitate goal setting at each visit; 4) record health information; and 5) contact patients' GPs if patients were symptomatic or had critical blood glucose or blood pressure measurements. | Improved adherence to lifestyle modifications and outcomes such as improved A1C and weight control in DEP patients. |
| Aridi, 2014 [^31^](https://www.zotero.org/google-docs/?nYZDRM)  Kenya | Generalist, Community Health Workers | 1) Hygiene and sanitation, 2) family planning, 3) MCH,4) NCD, 5) communicable disease (TBC, malaria), and 6) HIV | 20 households with no remuneration.  100-150 households with remuneration ($45 per month)  2-3 hours per week  (no remuneration)  4 hours per day (with remuneration) | Government CHW: 1) carrying out health promotion on hygiene and sanitation; 2) community mobilisation to join family planning programs and HIV counselling; and 3) caring for people with HIV, which were tasks outside the MoH policy.  MPV CHW: The CHWs had the same task but placed more emphasis on the promotion of door-to-door voluntary counselling and testing (VCT) by trained counsellors. The management of TB patient care includes case finding, referring patients, and supervising the taking of medication. Assistance for pregnant women and toddler health, and management of malaria cases. | Both got a huge workload, they even got assignments outside MoH policy. Even though the CHWs were given incentives, they still could not reach the target because the burden was greater than those who were not given incentives. Will be fulfilled if they are employed full time. The obstacles are the same, that is, they cannot reach the targets because they have to earn income to meet family needs. |
| Smith, 2014 [^47^](https://www.zotero.org/google-docs/?qI1pOC)  Malawi | Generalist, Health Surveillance Assistants (HSA) | 1) MCH, 2) communicable Disease ( TB), and 3) HIV | 1000 people per HSA | Promoted community participation in health care activities and provided disease surveillance services at the community level. | **Task overloading**: HSAs had to perform various tasks beyond their official job descriptions, leading to overloading and increased stress.  **Role ambiguity**: Many HSAs felt unclear about their roles, indicating a lack of clarity in their responsibilities.  **Training, compensation, and supervision**: Proper training, fair compensation, and effective supervision were identified as critical needs for HSAs to perform their duties effectively.  **Openness to role expansion**: Despite the challenges, some HSAs were willing to take on additional responsibilities, which may indicate their dedication and adaptability.  **Core task difficulty**: Growing workloads made it harder for HSAs to complete their core tasks, potentially impacting the quality of healthcare they could provide.  **Responsibility distribution**: Policymakers should carefully consider how to distribute HSAs' responsibilities between preventive and curative care, and between community-based and centre-based activities.  **Potential specialisation**: The potential for HSAs to specialise in certain areas of healthcare should also be taken into account to optimise their impact on the health system. |
| Kowitt, 2015 [^27^](https://www.zotero.org/google-docs/?cjgeoE)  Thailand | Generalist, Village Health Volunteer (VHV) | 1) MCH ,2) communicable disease, 3) mental health, 4) NCD (diabetes mellitus, 5) hypertension, obesity, 6) cancer, etc) | Not reported | 1) Conducting health education; 2) making referrals for the sick; 3) caring for the elderly; 4) monitoring food safety; 5) conducting disease surveillance; 6) helping to set health priorities and direct programs; and 7) providing social and emotional support to patients. | Not reported |
| Datiko, 2015 [^71^](https://www.zotero.org/google-docs/?WcNBSW)  Ethiopia | Specialist,  community health promoters (CHPs) | Tuberculosis | Not reported | Supporting Health Extension workers (HEWs) in their tasks (Collecting sputum, producing smears, supporting patient treatment seeking journey), and identifying possible TB cases | Community-based interventions increase access of vulnerable groups to diagnosis, treatment and treatment of tuberculosis. |
| LeFevre, 2015 [^48^](https://www.zotero.org/google-docs/?N1zCoC)  Tanzania | Specific, Maternal and neonatal child health community health workers (MNCH CHWs) | Maternal and neonatal child health | An average of 186 households or 967 people per MNCH CHW per catchment area. Average 39 home visits monthly for maternal and neonatal monitoring.  Provide MNCH services for 5 hours weekly, 39 visits monthly. Assuming a 20-day work month, CHWs would need to conduct almost two household visits per day. | Conducting surveillance of pregnancy and childbirth and providing counselling during three pregnancy home visits and six postpartum visits. | CHW remuneration and retention remain a concern, as 60% of CHWs feel overburdened due to other competing household and professional responsibilities to the extent that 14% indicate that they contemplated quitting. Younger CHWs were more likely to feel overburdened and less valued by community members. 80% of CHWs were dissatisfied with financial incentives provided  Incentive US$ 15.00 monthly  Workload (median): 120 households per CHWs, 3 days per week, 5 hours per day.  Meanwhile the target is: 5 households visit per day of 8 days work per month, or 480 households per year. |
| Kambarami, 2016 [^32^](https://www.zotero.org/google-docs/?IB0woy)  Zimbabwe | Generalist, Community health workers | 1) MCH, 2) Non Communicable Disease, 3) Communicable Disease, 4) sanitation and hygiene. | 100 households per CHW  4 hours per week | Provided basic health care treatment and health promotion education on a broad range of topics and reported monthly to the head nurse at their nearest primary health care facility. | From CHW perceptions of their work characteristics (female, unmarried, under 40 years old, from larger households, and of longer tenure), positive feedback and adequacy of work resources were the only factors significantly associated with more referrals.  The CHWs who were more satisfied with remuneration made fewer referrals. |
| Rabbani 2016 [^49^](https://www.zotero.org/google-docs/?CrAUF7)  Pakistan | Specific, Lady Health Workers (LWs) | Maternal, Newborn and Child Health | 100-150 households and approximately 1,000 people per LHW | Providing medical treatment and health education for maternal, newborn, and child (MNCH) Case detection, tracking, management, and follow-up of pneumonia and diarrhoea cases. | Supportive supervision, recognition, training, logistics, and salaries are community and health system motivating factors for lady health supervisors. Lady health supervisors are motivated by both their role in providing supportive supervision to lady health workers and by the supervisory support received from their coordinators and managers. Family support, autonomy, and altruism are individual level motivating factors |
| Chin-Quee, 2016 [[61](https://www.zotero.org/google-docs/?SIcR5L)  Rwanda[]](https://www.zotero.org/google-docs/?Xwa8cp) | Generalist, Community Health Workers | 1) MCH,2) family planning, 3) TB, 4) HIV/AIDS, 5) malaria, 6) nutritions, 7) sanitation and home hygiene. | 40 clients per week  20 hours per week | There are 3 CHW per village. One CHW is in charge of maternal and child health and the other two comprise a binome. Binomes–a male and female pair of CHWs in each village–are tasked with providing community-based integrated management of child illness; growth monitoring of infants and children; family planning counselling, referral and condom provision; directly observed treatment of tuberculosis; rapid tests for malaria diagnosis; nutrition counselling; counselling on sanitation and home hygiene; as well as HIV/AIDS prevention and support for people living with HIV/AIDS | The CHWs felt they had too many responsibilities, not enough time for family or themselves, and a lack of supplies or commodities.  Meanwhile, the clients were satisfied with the services provided by CHWs, especially counselling about family planning, nutrition, and sanitation. Most clients reported that the quality of service for some CHWs had improved and remained constant over the past year. |
| Maravilla, 2016 [^38^](https://www.zotero.org/google-docs/?5F9GYT)  Australia | Specialist, Community health worker (CHW) | Repeat pregnancies and births among adolescents | 10 - 15 adolescents per CHW  Frequency ranging from 1-2 times weekly to 2-4 times monthly | Home visitation | CHWs’ involvement with adolescents led to a 30% reduction in the risk of repealed birth among teenagers 2 years after their first pregnancy while no significant reduction was found regarding repeated pregnancy. |
| Siekmans, 2017 [^88^](https://www.zotero.org/google-docs/?Ql3WO8)  Liberia | Specialist, Community Health Workers | communicable disease (1) pneumonia and 2) diarrhoea when ebola outbreak) | Not reported | Treating and referring children who have malaria and pneumonia. In the midst of the Ebola crisis, CHWs were charged with informing the public about Ebola prevention, making house-to-house visits, distributing health information at community meetings, and engaging in active case detection to support the Ebola task force. | After receiving training, CHWs can assist in providing healthcare during an Ebola outbreak. As a result, more children with diarrhoea and malaria received treatment throughout the pandemic. |
| Khetan, 2018 [^44^](https://www.zotero.org/google-docs/?AXIaQ2)  India | Specialist, Community Health Workers | Non communicable disease | 120 participants per CHW (25 HH)  40-60 hours per month | 1) Provided home-based counselling to people with hypertension, diabetes, and smoking problems; and 2) visited patients every 2 months. | A population-based strategy of integrated risk factor management through community health workers led to improved systolic blood pressure in hypertension, an inconclusive effect on fasting blood glucose in diabetes, and no demonstrable effect on smoking. |
| Wroe, 2021[^28^](https://www.zotero.org/google-docs/?w5oz5w)  Malawi | Generalist, Community Health Workers | Comprehensive household model:  1) TB, 2) HIV, 3) NCDs, 4) nutrition, 5) family planning, 6) STIs  Pre intervention: HIV and TB | 20-40 (average 38) household/ month | 1) home-based screening (STIs, TB, HIV, and paediatric malnutrition) monthly; 2) provided education; 3) provided support for linkage to care for symptomatic clients or women who may be pregnant; 4) provided accompaniment for patients in care (adherence support, psychosocial support, tracking of missed patient visits). | Improvements were observed in antenatal care attendance and rates of default from chronic care with no evidence of a change in the default rate from HIV care, which was already covered by the previous programme with very strong results.  Evidence of improvement in paediatric malnutrition and TB case finding were not detected. |
| Whidden, 2018 [^55^](https://www.zotero.org/google-docs/?6o7F7F)  Mali | Specialist, Community Health Workers | Maternal, newborn, and child health | Not reported  2 hours per day | 1) conducted proactive case detection by door-to-door home visits; 2) provided doorstep counselling, evaluation, diagnostics, treatment, and referral to appropriate health facilities; and 3) followed up for at least two hours per day.  CHWs were required to be available at home or by phone for consultation at any time | Dedicated monthly supervision and personalised feedback using performance dashboards can increase CHW productivity  All CHWs were compensated with a monthly salary of 40000 FCFA (approx. US$ 70) plus 1000 FCFA (approx. US$ 2) in mobile phone communication credit for this part-time work |
| Ludwick, 2018 [^23^](https://www.zotero.org/google-docs/?BLYD5a) | Generalist, Community Health Workers | Maternal and Child Health and environmental health | Not reported | Provided health promotion about maternal and child health and sanitation. | Four components, supportive supervision, good relationship with other healthcare workers, peer support, and retention and incentive structures, received the lowest overall scores. CHW team performance related issues of absentee supervisors, referral system challengers, and lack of engagement/ respect by health workers |
| Shelley, 2018 [^29^](https://www.zotero.org/google-docs/?9o1Rt2),  Tanzania | Specific, Community Health Worker | 1) Maternal, newborn and child health (MNCH), and 2) HIV | 15 - 100 households per CHW | HIV CHWs roles include: 1) tracking patients who missed appointments; 2) linking patients to HIV care; 3) providing psychosocial support and palliative care; 4) promoting anti-retroviral treatment adherence; 5) mobilising the community; 6) offering home-based HIV counselling and testing; and 7) organising economic strengthening groups.  MNCH CHWs roles include: 1) visiting at least three times during pregnancy and seven times postpartum; 2) three home visits within the first week following birth and recurrent follow-up visits at 3 and 5 weeks, 2 and 5 months, and quarterly until the child turns five; and 3) providing health promotion topics covering a range of issues (HIV and MNCH). | The average of double-role CHW home visits was higher than that of single-role CHW visits, but not statistically significant. |
| Van Boetzelaer, 2019 [^40^](https://www.zotero.org/google-docs/?ttkOkr)  Sudan | Specific, Community-based distributors (CBD) | Child nutrition | 4 households per week | 1) Welcoming the caregiver; 2) assessing for danger signs, including bilateral pitting oedema; 3) taking the child's MUAC measurement; 4) conducting an appetite test; 5) determining the weekly RUTF dosage based on the child's weight; 6) administering medication (Amoxicillin for week 1, Albendazole for week 2); 7) filling out the patient register; 8) counselling the caregiver; 9) child progress monitoring and child discharge. | This study shows that low‐literate CBDs in South Sudan were able to follow a simplified treatment protocol for uncomplicated SAM with high accuracy using low‐literacy‐ adapted tools, showing promise for increasing access to acute malnutrition treatment in remote communities. |
| Karuga, 2019 [^69^](https://www.zotero.org/google-docs/?6sIjW8)  Kenya |  |  |  | Provided health promotion, health education, basic curative and referral services | Six months after the intervention, we observed that supervisors had shifted the supervision approach from being controlling and administrative to coaching, mentorship and problem solving. Changes in the frequency of supervision were found in Kitui only, whereby significant decreases in group supervision were met with increases in accompanied home visit supervision. Supervisors and CHVs reported in the intervention was helpful and it responded to capacity gaps in supervision of CHVs. |
| Musoke, 2019 [^34^](https://www.zotero.org/google-docs/?l10P4W)  Uganda | Generalist, Village Health Teams (VHTs) | 1) water, sanitation and hygiene; 2) malaria, 3) pneumonia, 4) diarrhoea, 5) non-communicable diseases; 6) maternal and child health | Not reported | 1) Community mobilisation, 2) health promotion at household and community levels, and 3) linking the population with health facilities including referral of patients. Where CHWs are functional, they have contributed to: raising health awareness; increased demand and utilisation of health services; and decongestants at health facilities as they treat minor childhood illnesses of malaria, diarrhoea and pneumonia. | The majority of CHWs 292 (98%) felt competent in their roles including diagnosis, record keeping and referral of sick children.  By the end of the project, the CHWs had conducted 40,213 household visits, carried out health education sessions with 127,011 community members, and treated 19,387 children under 5 years of age. From the project evaluation, which used both quantitative and qualitative methods, 98% of the CHWs reported having improved competence in performance of their roles. In addition, the CHWs were highly motivated to do their work. The motorcycles were instrumental in supporting the work of CHW coordinators including monthly collection of reports and distribution of medicines.  CHW got non-financial incentives of t-shirts, umbrellas, gum boots and certificates to all CHWs), and solar equipment to 75 of them. Monthly mobile telephone credit was provided to the coordinators of CHW |
| Kok, 2019 [^24^](https://www.zotero.org/google-docs/?TlJwZA)  Tanzania | Specific, Community-based mobilizers (CBMs) | 1) Family planning, 2) post-abortion care, 3) cervical cancer | Not reported  1-4 days per month | Raising awareness, generating demand, and providing referral to people for healthcare services. | Most CBMs were motivated to conduct their work because of an intrinsic desire to serve their community. The most mentioned extrinsic motivational factors were recognition from the community and supervisors, monthly allowance, availability of supporting materials and identification, training, supervision and feedback on performance. Recommendations for improvement were translated into the DCE. Incentive attributes that were found to be significant in DCE analysis (p < 0.05), in preference order, were carrying an ID card, bi-monthly training, supervision conducted via both monthly meetings at clinics and visits from the head office, and a monthly flat rate remuneration (over pay for performance). |
| Oluwole, 2019 [^62^](https://www.zotero.org/google-docs/?zlHIhB)  Nigeria | Specific, Community Drug Distributors (CDDs) | Neglected Tropical Diseases (NTDs) | Not reported | Medicine administration, but also in community engagement which contributes to ensuring equitable access to and acceptability of medicines. | The problems and solutions identified by frontline implementers were organised into three broad themes: technical support, social support and incentives. Areas identified for technical support included training, supervision, human resource management and workload, equipment and resources and timing of mass administration of medicines (MAM) implementation. Social support needs were for more equitable drug distributor selection processes, effective community sensitisation mechanisms and being associated with the health system. Incentives identified were both non-financial and financial including receiving positive community feedback and recognition and monetary remuneration. |
| Goudge, 2020 [^50^](https://www.zotero.org/google-docs/?gw7uA0)  South Africa |  | 1) MNCH, 2) child nutrition, 3) TB, 4) gender based-violence, 5) family planning. | 150-250 households per CHW | offering services for health promotion, prevention, screening, and referral for a range of illnesses as well as chronic care for people who are elderly or disabled. | At household registration visits, the CHW asked half of the questions required. Respondents remembered 20%-25% of the health messages that CHW delivered from a visit in the last month, and half of the CHW, and collaboration with other clinic staff, were better with a senior nurse supervisor |
| John, 2020 [^35^](https://www.zotero.org/google-docs/?oHa0WF)  India | Specific, Community Health Workers | 1) Mother and Child Health, 2) Nutrition | 40 children and several pregnant women per CHW | Provided health education and promotion about mother and child health and child nutrition | The results showed that a high workload affects the CHW's duties but does not affect certain outcomes specifically. The majority of CHWs interviewed felt overburdened and stressed that the high workload lowered their motivation and affected job satisfaction, especially because they felt that the government was not paying them adequate wages for their efforts. |
| Chipukuma, 2020 [^33^](https://www.zotero.org/google-docs/?nF93HW)  India | Specific, Community Health Workers | Malaria | 11.367 households per 36 CHWs or around 316 households per CHW | Carrying out malaria prevention and control activities | CHWs felt burnout because the number of targets was too large compared to the number of CHWs, which is limited, and also because they had other jobs. |
| Raven, 2020 [^64^](https://www.zotero.org/google-docs/?YRKWMm)  Africa Region: Sierra Leone, Liberia, Republic of Congo. | Generalist, Community Health Workers | 1) Reproductive, 2) MNCH, 3) child health (pneumonia) and 4) diarrhoea | Not reported | Providing preventive, curative, promotive, rehabilitative and palliative services as well as surveillance. | **Education and literacy levels**: In fragile settings, community members may lack the necessary literacy levels required for CHW roles, making it difficult to effectively engage and communicate with the community. This can hinder the selection and performance of CHWs. **Community-level policy preferences**: To address the issue of CHW selection, policies should be discussed at the community level to ensure they align with the realities and needs of the local population.  **Ongoing training for CHWs**: A comprehensive support system, including supervision, community support, regular supply provision, performance rewards, and timely remuneration, is essential to retain and enhance the performance of CHWs.  **Challenges with support and supplies**: However, there are challenges with supervision, scarcity of supplies, inadequate community recognition, and unfulfilled promises regarding allowances, which can negatively affect CHWs' motivation and performance.  **Clear community and incentives**: Clear and transparent communication about incentives and allowances with both facility staff and communities is crucial to maintain trust and motivation among CHWs.  **Workload concerns**: Managers in all settings recognize workload as a key reason for ineffective supervision structures. Managing workload is essential to ensure CHWs can perform their tasks effectively. |
| Gottert, 2021, [^81^](https://www.zotero.org/google-docs/?cZN5RN)  Mali and Bangladesh | Mali: Generalist, Community Health Workers (CHWs)  Bangladesh: Specific, Family Welfare Assistants (FWAs) | CHWs in Mali: 1) WASH, 2) children, nutrition,3) family planning, 4) HIV/AIDS, 5) TB, 6) Malaria, 7) pregnant women)    FWAs in Bangladesh: family planning | Not reported | Mali CHWs: 1) behaviour change communication; 2) antenatal/postnatal home visits; 3) management of sick children; 4) providing education on water, sanitation, and hygiene (WASH); 5) nutrition; 6) family planning; 7) HIV/AIDS; 8) tuberculosis; and 9) malaria.    Bangladesh FWAs: 1) listing and mobilising pregnant women for services; 2) counselling eligible couples on contraceptive methods and side effects; 3) providing pills, condoms, and the second dose of injectable contraception; 4) identifying and referring couples for clinical contraception; and 5) assisting in immunizations. | 68.4% CHWs in Bangladesh and 90.8% CHWs in Mali were satisfied (motivation) with the number of hours they work in a typical day.  Both Mali and Bangladeshi CHWs said that the remuneration was insufficient. The FWAs felt they had too many responsibilities. |
| Ashebir, 2021, [^89^](https://www.zotero.org/google-docs/?iC5CS8)  Ethiopia | Women's Development Group | 1) Maternal, neonatal, and Child Health | Not reported | Provided a community link to the health extension services with maternal, neonatal, and child healthcare to households. | The current WDG leaders defined their work broader, i.e., as community development agents who also were responsible for activities outside the health sector. These ambiguities have led to poor management of the group. |
| Kawade, 2021, [^90^](https://www.zotero.org/google-docs/?plnMBx)  India | Generalist, Accredited Social Health Activists (ASHA) | 1) MCH, 2) NCD | 20-40 visitation per week, or serving population approximately 800-1200 per ASHA  Up to 20 hours per week | Provided education on family planning, antenatal care, and care during childbirth. Screening, early detection, referral, and community mobilisation of non-communicable diseases (NCDs). They sometimes did non-health-related tasks that might take priority over health-related issues. | ASHAs were struggling to balance their significant ASHA work and domestic tasks. Nevertheless, they were proud of their role as CHWs and willing to take on new activities with the hope that there would be an increase in incentives from INR 1500 to INR 2000–5000. |
| Ndambo, 2022 [^41^](https://www.zotero.org/google-docs/?zi7abw)  Malawi | Generalist, Community Health Workers | 1) MNCH, 2) child nutrition, 3) communicable disease (TB), 4) HIV, 5) non communicable disease, and 6) family planning | 20-40 households per CHW | The primary roles of the CHW are to monitor the health of their assigned households, conduct health education activities, screen and link community members to essential health services at the health facility, collect data for reporting, and support patients and community members in their assigned households. The CHW’s roles are summarised into five main categories, namely; 1) monitor/screen; 2) educate; 3) collect data and report; 4) accompany/refer; and 5) support. | Themes of complex intrinsic and extrinsic factors were generated from the perspectives of the CHWs in the focus group discussions. Study results indicate that enabling factors are primarily intrinsic factors such as positive patient outcomes, community respect, and recognition by the formal health care system but can lead to the challenge of increased scope and workload. Extrinsic factors can provide challenges, including an increased scope and workload from original expectations, lack of resources to utilise in their work, and rugged geography. However, a positive work environment through supportive relationships between CHWs and supervisors enables the CHWs. |
| Musoke, 2021 [^36^](https://www.zotero.org/google-docs/?aCL4Ua)  Uganda | Specific, Community Health Workers | 1) Childhood illnesses, 2) maternal and child health, and 3) HIV/AIDS. | Not reported | CHWs engage with communities to identify local health problems and needs, mobilise them for health interventions, and refer and link them to health providers including follow-up. CHWs also collect and maintain records, conduct home visits, treat children aged under 5 years, and provide basic health education. | The availability of incentives (financial and nonfinancial), enhanced transportation, manageable workload, and adequate support and engagement were important elements for CHW motivation. Ensuring adequate numbers and distribution of CHWs in all villages would reduce their workload as well as distances travelled to communities. |
| Roy 2021 [^11^](https://www.zotero.org/google-docs/?FNkYzQ)  Bangladesh | Family Welfare Assistants (FWA); Health Assistants (HA); and Community Health Care Practitioners (CHCP) | 1) Family planning, 2) immunisation, 3) disease surveillance, etc. | 20,000-120,000 people per CHW  5-6 days a week | FWAs provide counselling and promotion of family planning services under the Directorate General of Family Planning (DGFP); HAs support the Expanded Programme on Immunization (EPI), disease surveillance, and provide other primary health care services under the Directorate General of Health Services (DGHS); and CHCPs provide preventive and primary health care at the community clinics (CCs). | The CHWs felt that their workload had become quite burdensome, which had significantly affected their motivation and morale. Given the lack of an adequate CHW-to-population ratio, the existing CHWs were often overstretched and were responsible for supporting more people than originally intended, which adversely affected CHWs and raised concerns about their ability to work effectively.  This study highlights that institutionalisation of CHWs without adequate and sustained support for continued training, compensation, supervision, access to working tools, and recognition is insufficient to drive change. |
| Dam 2022 [^73^](https://www.zotero.org/google-docs/?FkaLmC)  Multiple countries | Specific, Community Health Workers | Tuberculosis | Not reported | Active case finding: 86% of projects had CHWs complete community outreach and verbal screening; 72% of projects had CHWs collect and transport sputum; 70% of projects had CHWs link TB treatment to patient; 50% had CHWs conduct treatment counselling to patients; 8% of projects had CHWs conduct HIV testing. | CHW interventions overall had a large positive impact on yielding incremental TB notifications in intervention areas. The CHW model factors more likely to influence project outcomes are the training method and setting, supervisory models, and integration into the existing health system. |
| Kawakatsu 2022 [^52^](https://www.zotero.org/google-docs/?Kmoz4W)  Kenya | Generalist, community Health workers | 1) MCH, 2) communicable disease, 3) non communicable disease. | 100 households per CHWs | Regular household visits for the purpose of (i) providing health education sessions at each household; (ii) identifying women having danger signs during pregnancy and other households members’ health problems; (iii) referring them to the health facility linked to the Community Unit; (iv) monitoring patients’ recovery process at households; and (v) collecting updated households’ sociodemographic and health data. They were not authorised to provide household members with any treatments | The refresher training significantly improved their health knowledge, while financial incentive enhanced the level of their job satisfaction. The combinations of regular refresher training and other intervention(s) are recommended as the effective interventions in improving and further sustaining CHWs’ performance. |
| Ishizumi 2021 [^91^](https://www.zotero.org/google-docs/?PYzm4j)  Sierra Leone | Generalist, Community Health Workers | 1) MCH, 2) malaria prevention, 3) hygiene, and sanitation. | Not reported | Daily household visits, referral or escort to local healthcare facilities, support for MCH activities in areas, such as community education and outreach, supporting pregnant women and sick children, distributing bed nets, providing community education on bed net usage as well as water, sanitation, and hygiene (WASH) issues, performing rapid diagnostic tests, distributing medications for treatment, actively promoted immunisation via community education and reminders. They described playing an active role in defaulter tracing, which usually involved scanning of child health cards during household visits or conversations with defaulters to promote catch-up vaccination, rapid diagnostic tests for malaria and referral of defaulted children to health facilities for catchup vaccination. | 1) pride, compassion, recognition, and personal benefits are important motivating factors to keep working as CHWs; 2) divers health responsibilities and competing priorities result in overburdening of CHWs; 3) health system- and community - level barriers negatively affect CHWs’ activities and motivation; 4) CHWs use context-specific strategies to address challenges in their work but require support.  It is possible that CWHs performance may be hindered by having an unrealistic workload under a volunteer scheme. |
| Pandya, 2022 [^45^](https://www.zotero.org/google-docs/?BaX5kx)  Uganda | Generalist, Village Health Workers (VHWs) | 1) Childhood illnesses; 2) family planning; 3) antenatal care; 4) water, sanitation, and hygiene | 25 - 30 households per VHW | They are responsible for providing a range of services at the community and household levels, which includes health promotion and education, mobilisation of communities for utilisation of health services, case management for specific infectious diseases, referrals to health facilities and follow-ups, and the distribution of health commodities. | Introducing a new paid CHW, without addressing issues with the existing VHT CHW, could backfire.  The VHWs were unable to carry out their roles because they did not receive a salary or allowance to reimburse transportation costs. They want recognition from health workers such as not returning the referrals they bring, getting certification, and comprehensive training in the areas where they routinely provide services. |
| Toney, 2022 [^74^](https://www.zotero.org/google-docs/?Ltalc5)  Nebraska | Generalist, Community Health Workers | Primary Health Care, MCH | Not reported | Interpretation, case management, health screenings, referrals, and insurance applications, home visit. | CHWs often felt powerless when clinics did not accept patients without insurance or they were immigrants.  CHWs acknowledged the difficulties in accessing transportation for their clients and described their frustrations with the limits placed on transportation.  CHWs collectively voice that the shortage of staff, increased workload and caseloads, and overtime makes it difficult to provide quality care to their clients |
| Roy, 2022 [^67^](https://www.zotero.org/google-docs/?5uv7I4)  Bangladesh | Generalist, Family Welfare Assistants (FWAs) and Health Assistants (HAs) | FWAs: family planning  HAs support the Expanded Programme on Immunization (EPI) and disease surveillance and provide other primary health care services | Not reported  At least 2 days per week in fixed facilities | FWA: home visits to search for newly married couples, distribute pills and condoms, provide RTI and STI counselling; administer second and subsequent injectables; identify pregnant women and conduct counselling for ANC visit; act as community skilled birth attendant if trained.  HAs: vaccinated mothers and children under EPI program; conducted home visits for counselling on NCD and WASH activities; diseases surveillance.  Both: implement maternal and newborn health, FP, and nutrition activities in community; advise on postpartum FP; conduct home visit for postnatal and newborn care; participate in satellite clinics to arrange immunizations; implement vitamin A campaign in the community; and conduct record keeping and reporting. During pandemic they had new responsibilities related to COVID-19 prevention, treatment, referring, and reporting. | New responsibilities increased FWAs’ and HAs’ workload and detracted from routine service provision, exacerbating the ongoing challenge of a CHW workforce shortage in Bangladesh.  Qualitatively, CHWs expressed discontent with the lack of incentives accompanying the high expectations to work and undertake new responsibilities.  The biggest challenge was fear of contact with infected people, not having enough medicines in facilities, hospitals being overwhelmed by patients with COVID-19, and transport to facilities being unavailable. |
| O'Donovan, 2022 [^42^](https://www.zotero.org/google-docs/?cVDhRX)  Uganda | Generalist, Community Health Workers (CHW) | 1) Maternal and child health, 2) malaria, 3) pneumonia, 4) ear disease, 5) hypertension. | 28 -120 households per CHW | Addressing common childhood and maternal challenges, such as malaria and pneumonia, ear disease and hypertension through health education, promotion and carrying out household visits. | Supervision is perceived both as a means of motivating CHWs and facilitating ongoing training, as well as a way of holding CHWs accountable for their work. |
| Gadsden, 2022 [^53^](https://www.zotero.org/google-docs/?BUAnZ0)  Indonesia | Generalist, Kader | 1) Mother and child health, 2) Hypertension, and 3) Diabetes, 4) child nutrition | Not reported.  Less than 2 - more than 4 hours a week. (Flexible working hour) | Assisting village midwives to provide activities including health and nutrition counselling, immunisation campaigns, monitoring and screening activities for diabetes and hypertension, and maternal and child healthcare. Screening community members for cardiovascular risk using a tablet-based application. | The most important influence on choice of job was a low monthly financial benefit followed by recognition in the form of a performance feedback report. |

Table S3 (b). Supervision Mechanism of Community Health Workers

| **Author Year Location** | **CHW name**  **Health Issues** | **Supervisor** | **Supervision Process** | **Supervision Output** |
| --- | --- | --- | --- | --- |
| Kaphle 2016, [^39^](https://www.zotero.org/google-docs/?1s1L2s) India | Community nutrition experts.  Mother and child health | The project coordinator, the community nutrition experts’ immediate supervisors, and the district supervisor. | The community nutrition experts received supportive supervision, recognition, performance feedback, and training in problem areas with their app. They contributed to the program by discussing challenges and issues, which were escalated to the program coordinator for better intra-organizational communication. Weekly calls allowed for relaying feedback on performance metrics. Phone calls provided a means to address work-related issues, personal needs, mistraining, or technical difficulties with the mobile app, promptly resolved by district supervisors. | Positive and significant impacts on duration of counselling, whereas case activity and number of form submissions did not show significant improvements as a result of the intervention. The study found a moderate to large effect (Glass’s delta=0.97, *P*=.004) of providing performance feedback on counselling times in the initial 6 weeks.  Calls can improve performance due to elements of supportive supervision included in the calls encouraging CHW motivation. |
| Kawasaki 2015, [^15^](https://www.zotero.org/google-docs/?Bq0cBf)  Brazil | Maternal and Child Health | Healthcare workers. | The supervision mechanism is face-to-face meetings using a manual reporting method. At the monthly meeting, health workers and CHWs refreshed the materials presented during the training, therefore CHWs will continue to be able to carry out quality services. It is not clear how many CHWs are supervised by one supervisor | Supervision is running effectively. The article explains that prior to the existence of the project, health workers at the *puskesmas* and CHW did not collaborate. However, after the project was established, health workers were asked to be involved in refreshing the information provided during the training so that collaboration between health workers and CHWs was established. |
| Hill 2014, [^58^](https://www.zotero.org/google-docs/?V1BSGz)  Multiple countries | N/A | Most studies had health workers as the supervisor of the CHWs. | Varies. Most studies conduct training from supervisors. | 1. Supervision frequency: increasing frequency alone does not necessarily lead to increased effectiveness. Studies show variative results on association between supervision frequency and CHW performance. 2. Supportive/Facilitative supervision package: considered best practice, brings significant changes in CHWs performance or program outcome; however requires some support and enabler factors. 3. Peer supervision: studies show positive impacts on CHWs performance. Provide a career pathway and a more affordable alternative. 4. Group supervision: as effective as standard supervision. May be appropriate if supervisors are far from CHWs. 5. Community supervision: modest impact and quality. 6. Self-assessment: All studies reported (at least) improvements in communication and interpersonal skills, better quality of care/services, and higher client satisfaction. 7. Checklists 8. Quality Assurance and problem solving: all had some positive impacts, but required additional resources. 9. Supervisor training: results vary (some show statistically significant improvements in CHWs), might due to differences in types of supervision.   Conclusion: improving supervision quality has a greater impact than increasing frequency of supervision alone. Supportive supervision packages, community monitoring and quality improvement/problem-solving approaches show the most promise. |
| Rodriguez 2015, [^8^](https://www.zotero.org/google-docs/?pjZ4pl)  Malawi | CHWs,  Mother and Child Health | Environmental Health Officer | CHWs make periodic visits to health facilities to work under trained professionals and may be directly supervised by supervisors. | Supervision is not running effectively, the problem is that environmental health officers (supervisors) are not trained in case management. Even though the supervision was not running optimally, there were partners who assisted CHW. Partners play a role in transferring knowledge to the CHWs. |
| Khetan 2018, [^44^](https://www.zotero.org/google-docs/?3m4mro)  India | CHWs,  Non Communicable Disease | The supervisor was selected from those who had worked earlier as CHWs for hypertension control in a pilot project. Supervisors had demonstrated leadership qualities and enjoyed teaching. They trained with the CHWs, with the addition of an extra day to every training block to teach their supervisory role.[^92^](https://www.zotero.org/google-docs/?DlIrkh) | Each supervisor is responsible for three CHW.  For every phase of the study, the supervisors randomly verified 10% of the work done by CHWs, following a standard protocol, which varied with the project phase. For example, for a diabetes visit, the blood glucose check was verified, knowledge level of the patient was assessed through questions and feedback was sought from the patient. The project manager, with the help of the study investigators, created a bimonthly list of participants who had to be verified by each supervisor. The supervision process was used to provide feedback to CHWs and identify areas of improvement. The supervisors usually provided extra support to CHWs who needed help in a particular area, often accompanying them on visits and discussing individual patients with them. While there were no requirements put in place for the study investigators to visit the patients, one of the study investigators visited patients on an ad hoc basis to understand how the intervention was working. The CHWs also maintained contact with a study investigator, discussing issues as they arose. The study investigators sought feedback from CHWs on their interaction with supervisors, and issues between supervisors and CHWs were addressed in a bidirectional fashion. | The impact of CHWs performance in the intervention group is higher and significant than the control group. The control group didn’t get the supervision like the intervention group. |
| Ngugi 2018, [^21^](https://www.zotero.org/google-docs/?KJfFll)  Kenya | CHWs,  1) Mother and Child Health, 2) Communicable Disease | First group are supervised by Community health extension worker (CHEW), and the second group with  Community Health Committees (CHCs). | - Supervision time is only categorised as : None, 1 times, 2 times, 3 times, >3 times in month - Method : The CHCs would conduct monthly supervision to review their reports and forward their reports to CHEWs if the CHWs lived far from the health facility. Sometimes the CHCs validate their reports by accompanying them during household visits. - Report submitted to household Community Health Information System (CHIS) | The CHWs, however, felt that CHCs were often overwhelmed by the task and suggested that the village elders be included to support in some areas. The participants felt that identification cards (given to them) had improved their recognition in the community.  The CHWs, however, felt that CHCs were often overwhelmed by the task and suggested that the village elders be included to support in some areas.  The inclusion of the Public Health Officers (PHOs) and CHEWs as part of the supervision team also enhanced their stature as was pointed out by active CHWs. They also advocated for the CHC to receive some token (allowance) for the work they do |
| Bhattacharji 1986, [^46^](https://www.zotero.org/google-docs/?u3dvec) India | Mother and Child Health | Supervision is carried out by a health aide (health assistant) who is selected by the health centre staff who has the minimum educational qualification of the school final exam. Its training is much more detailed than that of part-time community health workers (PTCHWs) and lasts one year, of which six months are in the field. The Health Assistant is responsible for maintaining all basic data of their community with the help of PTCHW. There are 15 health workers and each is in charge of three to four PTCHWs. | Once every 2 weeks, health assistants come to every village to provide supervision to PTCHWs. They supervise PTCHWs at work and check their medications and the number of visits PTCHWs make. Seeing PTCHW providing home health education, screening for high risks etc. As well as correcting and encouraging PTCHWs in various aspects of their work. If the PTCHW has treated a patient and has a question, the volunteer often uses this opportunity to raise it and the problem is resolved with the team. PTCHW was encouraged to make decisions and made to feel that CHW would be supported in everything he did. Of course, any errors were pointed out and corrected.  All PTCHWs meet once every two weeks, together with the doctor in charge and the PHN. At these meetings they share their experiences, especially their difficulties, and any doubts and questions they may have. The staff also has a schedule plan for current reviewed topics. The concept of a team approach is emphasised and much effort is made to build good relations among the team members.  All decisions taken are forwarded to the health workers and PTCHW by PHN and doctors. Two-way flow of information is emphasised, and efforts are made to ensure that PTCHWs and health workers are provided with feedback on information and analysis. They are therefore actively involved in the evaluation process and are free to suggest changes, which they do frequently | Up to the time this research was conducted, PTCHWs accepted corrections and learned quickly from their mistakes. So far there have been no instances of the wrong drug being prescribed. A frequent error is PTCHW's assessment of their ability to handle a given situation, such as a difficult birth, and at what point to make a referral.  Although the project team stressed that support and supervision were essential for the effective functioning of the PTCHW, it appeared that the PTCHWs that received intensive supervision did not perform significantly better than those that received less. However, this study does not explain what the situation would be if there was no supervision at all after training. |
| Assegaai 2019, [^61^](https://www.zotero.org/google-docs/?aCpkXr)  South Africa | Mother and Child Health, Chronic Disease | Supervisors are retired nurses (team leaders). One nurse supervises 6 CHWs. | Supervise directly in the field. For example, when CHW is attending training, team leaders attend training to familiarise themselves with the curriculum and observe how CHW performs in the training. In some cases, the team leaders are trainers for the CHW. It is the responsibility of the team leaders to provide in-service training to CHWs as part of an effort to improve CHWs' clinical and technical skills, as well as to motivate CHWs to continue increasing their capacity.    Report form: no clear mechanism, reporting is often informal. | The report from CHW states that CHW's relationship with the team leaders is very good. The presence of a team leader at the household level makes it easier for clients to receive services and makes work easier.  The weakening of the performance and sustainability of CHWs in South Africa is due to weak design and implementation of monitoring systems. Official policy documents and guidelines are inadequately developed and misaligned, both in terms of scope and in providing firm guidance on WBOT oversight. |
| Hennein 2022, [^14^](https://www.zotero.org/google-docs/?uK9qEP)  Uganda | Communicable Disease TB | Supervisor: Research Team, and weekly meetings are also attended by health workers | Supervisor is conducted every week inFriday morning.  The site visit is carried out by holding a meeting. Research Staff Staff provide weekly performance reports to CHW that include key indicators for conducting TB contact tracing for each site. CHWs may review their own individual reports to monitor their performance from time to time. CHW received instructions on what to do, which parts had not been implemented optimally, were there any steps that were missed. Experience sharing is also carried out at this weekly meeting, so that the CHWs can also provide suggestions. WA groups are also created when there are CHWs who need to communicate with supervisors | CHW stated that the reports and feedback provided had enabled them to work on the right track. And the supervisor is always there to help if CHW encounters problems. The article does not explain in detail the final impact on program outcomes, but the results of interviews with CHW show that having CoP interventions for CHWs makes them do their job better and get solutions to the problems they encounter in the field (regarding TB contact tracing). |
| Kambarami 2016, [^32^](https://www.zotero.org/google-docs/?PvH0at)  Zimbabwe | MCH, NCD, CD | Thirty-two SHINE nurse supervisors provided constructive feedback and supervision support for the 342 CHWs. | - Supervisors met once a month with CHWs for group meetings (approximately 11 CHWs per supervisor) to discuss concerns, troubleshoot, and review expectations. - Individual review meetings with CHWs were held in the field approximately once a month, and supervisors evaluated performance of specific tasks and provided additional support - Supervisors provided feedback to the CHW after each observation. | Supportive supervision was associated with higher scores, but operational supervision was associated with lower scores.  Higher task performance on pregnancy referrals (more referrals) was associated with more operational supervision. |
| Kelly 2001, [^59^](https://www.zotero.org/google-docs/?GWzYeq)  Kenya | MCH, Malaria | Health workers | According to supervisor reports, less than half of CHWs received one-to-one clinical supervision at a health facility in the past year. The frequency of supervisor contact with CHWs is not explained in the article. | Researchers found that supervisors of CHWs did not perform significantly better than CHWs in assessment and treatment. These results underscore the importance of monitoring supervisor skills and providing better support to supervisors. |
| Jerome 2010, [^43^](https://www.zotero.org/google-docs/?9cKPoa)  Haiti | HIV and TB care | Clinic staff | N/A | Some CHWs believe that there is sometimes a lack of recognition for their work by clinic-based staff, especially if a particular referral is not deemed appropriate. |
| Brenner 2011, [^37^](https://www.zotero.org/google-docs/?TwC6Xs)  Uganda | Child Health | Health centre staff. It is not explained how many CHWs are supervised by one supervisor. | Supervision is carried out every month. CHW is formed in teams to conduct monthly meetings with supervisors held during the study period. The monthly meeting includes 2- session refresher training and report presentation. | It was stated that the supervision mechanism was running effectively, but this article did not explain what the obstacles were and how the impact of these constraints on the output or achievement of the program. |
| Greenspan 2013, [^22^](https://www.zotero.org/google-docs/?PHx6P0)  Tanzania | Maternal and neonatal health program | N/A | Supervisors provide information and instruction, identify areas for improvement (such as filling out health cards more accurately), help solve problems, provide additional training, and encourage volunteerism. | In general, the system functions well. Some CHWs seek assistance from doctors at health centres, while others view supervision as a sign of underperformance. Supervisors play a role in motivating CHWs, leading to improved performance. |
| Collinsworth 2013, [^26^](https://www.zotero.org/google-docs/?rxwOUm)  USA | Diabetes Mellitus | Primary care providers (doctors) | Not explicitly explained, the impression taken is that the supervision took place at any time needed. | CHWs know their scope of role and when they should alert PCPs about potential patient problems. The CHWs said they felt comfortable interacting with PCPs as part of a care coordination team and worked with providers to provide patients with high-quality care. There is a 1.3% average reduction in A1C. |
| Munshi 2019, [^25^](https://www.zotero.org/google-docs/?wUiuxR)  South Africa | 1) MCH, 2) non-communicable disease, 3) communicable disease (TB); 4) HIV and 5) social issues | The supervisor of CHW is the CHWs team leader who is a professional nurse.  The team leader was supervised by the facility manager and district manager.  CHWs are known as the Ward-based Primary Healthcare Outreach Teams (WBPHCOTs).  The WBPHCOTs consist of 7-12 people. | Not explicitly explained, but the team leader supervised the CHW by site visit and phone call. | Team leader (CHW’s supervisor) couldn't fulfil all their WBPHCOT responsibilities because they have clinical responsibilities.  Having the team separated from the health facilities resulted in better relationships with facility-based staff including facility managers. The CHW reported directly to subdistrict and district managers, thus minimising the complication of dual lines of reporting to local government and provincial authorities, as was the case in our study.  The lack of transportation limited team leaders’ access to the team to conduct supervisory visits. |
| Smith 2014, [^47^](https://www.zotero.org/google-docs/?n9oxjS)  Malawi | MCH, NCD: HIV dan TB | Assistant Environmental Health Officers and Senior Health Surveillance Assistance (HSA) | Supervision is carried out by means of site visits | Supervision is not running effectively due to lack of integration and there are silos between departments. HSA is also not supervised properly because every program manager, program coordinator, will only supervise HSA if the program is in accordance with the scope handled by the supervisor, if outside the program they don't want to intervene. Due to ineffective oversight, this makes it difficult for HSAs to perform additional tasks and manage their workload. |
| Kowitt 2015, [^27^](https://www.zotero.org/google-docs/?NMAtfr)  Thailand | Health Issues | Public Health Officials (PHO) | There are regular meetings between Village Health Volunteer (VHV) and PHO, but the frequency is not explained | Several factors fostered a stronger relationship between VHV and PHO including PHO's appreciation of VHV's strengths and contributions, regular meetings between public health and VHV officials, involvement of VHV in program planning and implementation, and encouragement by public health officials for VHV to gain recognition from outside agencies, such as local and national governments. For communities where collaboration is limited, program planners and community leaders/organisers can use these factors to increase collaboration and promote a more participatory culture with input from VHV. |
| Datiko 2015, [^71^](https://www.zotero.org/google-docs/?X5PezG) Southern Ethiopia | TB | District Supervisor | The Health Extension Workers (HEWs) are regularly visited by supervisors twice a month and meet with TB providers in health centres once a month. The method of supervision by site visit. | Monthly update meetings are felt to be effective for solving problems and providing feedback between teams starting from HEW, Community Health Promoters (CHP) and technical labs. The greatest challenge for supervisors was the intensity of their workload coupled with the need to cover the large geographical area of their district, often with difficult terrain. |
| LeFevre 2015, [^48^](https://www.zotero.org/google-docs/?UITF7a) Tanzania | MNCH | MNCH CHWs were supervised by trained facility-based dispensary and health centre providers (enrolled nurses and/or clinical officers) through monthly supportive supervision visits and by Ministry of Health and Social Welfare (MoHSW) (regional and district) and Jhpiego staff on a quarterly basis. | - Supervision visits focused on a review of registers and reporting forms for data quality, activity planning, and a review of achievements and planning. - Facility-based providers also were reported to visit CHWs in the village a mean of once in 2 months - During both monthly and quarterly supervision visits, focus on checking the content of Health and Management Information System (HMIS) registers and knowledge assessments, feedback on work performance, work planning, and/or additional training | - Monthly and quarterly supervisory visits focused largely on HMIS registers. Despite this emphasis, inconsistencies were pervasive in CHW recordkeeping and nearly 25% of CHWs did not maintain MCH registers. - This raises concerns about the quality of register data and suggests that a review of register format and content may be warranted to reduce complexity and ease routine documentation of home visits by CHWs. - CHW attendance of supervisory visits was high, a factor which may be attributed to financial incentives disbursed, the amount of which corresponds to ~50% of the average the MNCH CHWs household income |
| Ndambo 2022, [^41^](https://www.zotero.org/google-docs/?nwAOuP)  Malawi | 1) Tuberculosis; 2) HIV; 3) STIs; 4) NCD; 5) family planning; 6) maternal and newborn health; 7) child health; and 8) malnutrition screening in children under 5 years | Senior CHW dan SS (Site Supervisor). | CHW seniors   - Time: Visits quarterly to homes. Apart from that, the Senior CHW also conducts unannounced checks by providing guidance and coaching to the assigned CHW every quarter. In addition, CHW Seniors also participate in monthly data reviews. - Method of Supervision: Carry out supervision at the village level. Each Senior CHW supervises 10-15 CHW people. - Form of Report: not explained. - Forms of Feedback: conduct guidance and coaching, apart from that the CHW Seniors also help solve problems in the field.   Site Supervisors (SS)   - Time: not specified - Means of supervision: SS supervises and guides SCHW and CHW through spot checks, data monitoring support, and surveillance meetings. - Report Form: Responsible for data aggregation, recording, and monthly reporting. - Forms of Feedback: conducting guidance and coaching. | Supervision is running less effectively. Some CHWs stated that he felt pressured to provide a report. |
| Maravilla 2016, [^38^](https://www.zotero.org/google-docs/?wvuunM)  Australia | Adolescent Repeat Pregnancies and Births | N/A | Supervisory support given in the studies often focused on data collection rather than performance evaluation and feedback | Findings from this review suggest these issues may not be as relevant as previously anticipated. CHWs without ongoing supervision effectively lowered repeat birth and repeat pregnancies, suggesting that the presence of a supervisor does not guarantee effective CHW management, but rather depends on the functions performed by the supervisors. |
| Revadi 2022, [^68^](https://www.zotero.org/google-docs/?Em3b4B)  Central India | N/A | CHWs Facilitators. Each CHW facilitator can monitor 20 CHWs | There is no detailed explanation. Based on FGD, supervision is carried out at certain moments such as when they have a survey or event. | CHWs performance cannot be ascribed completely to the CHW facilitators performance. However, their supportive score trends affect their performance. The barriers perceived by CHW facilitators on their CHWs performance were unmodifiable and required them to strongly motivate their CHWs and aid those in remote areas to achieve effective supervision. |
| Wroe 2021, [^28^](https://www.zotero.org/google-docs/?tmX77Q)  Malawi | Comprehensive household model (TB, HIV, NCDs, nutrition, family planning, STIs). Pre intervention: HIV and TB | Senior CHW per village (300 household) | Supervision meetings consist of CHW management meetings, site supervisor meetings, Senior CHW meetings, general meetings, and village-level CHW meetings. Additionally, SCHWs and site supervisors will conduct monthly supervision home visits, and monthly spot checks will be performed in order to: assess the performance of CHWs and SCHWs; identify areas of support; and identify gaps where the site supervisor or SCHW mentor can provide more clarity or support to CHWs. | 34-42% CHWs received supervision.  Site supervisors and HSAs used a structured checklist to identify any nutrition or TB clients that missed a visit or might need additional support, which was communicated to the CHW following those households. This allowed a much higher number of home visits to these clients than the HSA programme alone was capacitated for. Finally, management meetings to review staffing, data and performance were held at the district level. |
| Whidden 2018, [^55^](https://www.zotero.org/google-docs/?SMrxsG)  Mali | MCH | This randomised controlled trial used a dedicated cadre of CHW supervisors, recruited and trained for the exclusive purpose of supervising CHWs in the peri-urban area of Yirimadio in Bamako, Mali, each supervising 15 to 20 CHWs using a monthly dedicated supervisory strategy called 360 Supervision. | - CHWs in both study arms received monthly individual supervisory sessions (lasting approximately three hours) and weekly group supervisory sessions (lasting approximately two hours) from their dedicated CHW supervisor. - At weekly group sessions, which brought together CHWs in both intervention and control arms, the supervisor led discussion of the common challenges and potential solutions faced by CHWs. During the final group session of each month, the supervisor and CHWs agreed on a schedule of individual supervision sessions for the coming month. - Each CHW knew in advance the date and time, but not the location within his/her intervention zone at which the next individual supervision would take place - An individual monthly session of 360 Supervision included: (i) solicitation of patient perspectives of CHW care; (ii) direct observation of CHW doorstep care; and (iii) a one-on-one feedback discussion, with or without the CHW Performance Dashboard depending on treatment arm. - On the scheduled day of a CHWs individual supervision, the supervisor chose an area (different each month) within the CHWs zone to conduct home visits in the absence of the CHW for the purpose of soliciting patient perspectives of CHW care and verifying CHW reporting. - Supervisors interviewed the female head of household or her representative using a paper-based data collection form to record: knowledge of the CHW for improvement - After soliciting patient perspectives for at least one hour, the supervisor and CHW met for at least one hour of direct observation. At each home visit conducted by the CHW, the supervisor used another paper-based form to record his/her observations on: CHW behaviour/demeanour; questions asked and information provided; adherence to protocol; duration of the visit; maintenance of supplies; completion of patient care forms. Finally, the supervisor and CHW then sat together privately for approximately 45 minutes for a one-on-one feedback discussion of the CHWs strengths and areas for improvement, guided by another paper-based form and informed by the information recorded during the first two phases of individual supervision | The findings of this study suggest that the use of personalized performance feedback in dedicated monthly supervision can contribute positively and substantively to CHW performance improvement without compromising quality and timeliness of care. |
| Ludwick 2018, [^23^](https://www.zotero.org/google-docs/?ZXEumJ)  Uganda | MCH | Health centre workers. It is not explained how many CHWs are supervised | There is no detailed explanation regarding the supervision mechanism in this article. The group that stated that they often communicated with their supervisors stated that supervisors often contacted CHW when they experienced difficulties, guided them and supported all CHW tasks. Whereas the CHW group who did not often communicate with their supervisors said that they had only met a supervisor once since the training so they felt there was no teamwork with supervisors. | In this article, FGDs were conducted from groups with medium-high and low performance scores. 2 out of 4 groups that had low performance stated that they rarely had contact with supervisors which could make CHWs feel unwelcome, lack of technical support and mentoring. While those who have medium-high performance levels, communicate intensely with their supervisors. |
| Kok 2018,  [^60^](https://www.zotero.org/google-docs/?PBb4oI)  Multiple countries in Africa | CHWs conduct different areas of intervention in each country. CHWs mostly focus on maternal, newborn and child health. CHWs also conduct disease prevention and control, family health services, and environmental hygiene and sanitation such as in Kenya and Malawi. While in Mozambique, CHWs also intervene to diagnose and treat malaria, diarrhoea and chest infections. | CHW supervisors vary by country.   1. Ethiopia: health centre staff and Health Department 2. Kenya: Health worker 3. Malawi: senior CHWs and environmental health officers 4. Mozambique: health facility staff and district health directorates | This intervention is called "supportive supervision" which has characteristics such as problem-solving, shared responsibility and teamwork, cross-learning and skill-sharing, empowerment to self-assess or peer review, participation in decision-making, facilitating, mentoring, and coaching of the supervisor's role. These elements vary from country to country. Length of supervision training is about 5-6 days conducted by NGOs, Ministry of Health, Universities. The tools used in the supervision process are mostly supervision checklists. The types of supervision also vary by country.   1. Ethiopia: monthly individual supervision, monthly group supervision 2. Kenya: individual supervision, joint home visits; monthly group supervision 3. Malawi: Bi-weekly peer supervision, self-assessment, monthly group supervision 4. Mozambique: Monthly group supervision | (Quantitative analysis)   1. Job satisfaction: significantly increased after intervention in Mozambique; significantly decreased in Ethiopia. 2. Community commitment: no significant changes, relatively same after intervention. 3. Organisational commitment: significantly increased in Malawi; significantly decreased in Ethiopia. 4. Work conscientiousness: significantly increased in Malawi; significantly decreased in Ethiopia.   (Qualitative analysis)   1. Enhanced teamwork in Malawi and Kenya. 2. CHWs feeling more accountable in Malawi. 3. Feel empowered and included. |
| Assegaai 2019, [^56^](https://www.zotero.org/google-docs/?MBp81o) South Africa | MCH, NCD, communicable disease | Professional nurse. One nurse supervises an average of 3 WBOTs (21 CHWs) | Ideally, supervision is carried out in stages, namely the CHWs are joined in a team called WBOT and led by a puskesmas nurse who is called a team leader (TL) as a supervisor. TL itself is supervised by the head of the puskesmas. Then the head of the puskesmas is supervised by the local area manager (LAM) and the Camat. TL supervises the CHWs every day, the head of the puskesmas once a week, and the sub-district head once a month. | This supervision does not run in stages. Although most of the CHWs said that TL checked their work, a small number said the head of the puskesmas or LAM or NPK coordinator. There was no feedback from the head of the puskesmas to TL. Heads of puskesmas and sub-district heads do not play an active role in supervising WBOT work. Supervision at the middle level is not working. |
| VanBoetzelaer 2019, [^40^](https://www.zotero.org/google-docs/?ti0l12) Sudan | Child nutrition | CHW research officers and supervisors (not explained where they come from) | Supervision is carried out every biweekly by site visits | Supervision is running effectively. The CHW found the bi-weekly monitoring very helpful and allowed them to correct any mistakes, which was confirmed by CHW's research officers and supervisors on the spot. The training provided made the CHWs understand their duties better and made them know each other so as to influence member cooperation more effectively. There is an increase in the performance score of 2.0% for each supervision visit. However, because this study took a relatively small sample size, multivariate analysis was not performed. And fortnightly supervision visits still need to be explored again for their effectiveness in programs that are implemented on a large scale. |
| Karuga 2019, [^69^](https://www.zotero.org/google-docs/?eYZ3i4) Kenya | Community health approach, otherwise not specified | Community Health Extension Workers (CHEWs) have supervisory responsibilities over CHVs. CHEWs are formal public health sector employees. | CHEWs received training on: supportive roles (workers' welfare), administrative roles (performance related issues), educative roles (capacity building), problem solving skills, advocacy, prioritisation, identification of problems, developing actions. CHEWs were given supervision checklists as a guideline for every time they supervise CHVs.  Methods of supervision vary such as monthly meetings, accompanied household visits and by reviewing monthly reports. Supportive supervision means supervision sessions are not focused on correcting mistakes, but to support CHVs to perform better and solve problems. | CHEWs used monthly group supervision sessions to encourage, motivate, and also to mentor and coach their CHVs. CHVs felt motivated to supervise sessions to develop skills, they did not feel intimidated nor dictated. |
| Musoke 2019, [^34^](https://www.zotero.org/google-docs/?Y9ym7a) Uganda | Malaria, diarrhoea or pneumonia, health education | Supervisor is CHW coordinators with three motorcycles that were used to collect reports from CHWs as well as provide other supervisory support including distribution of medicines and other supplies | The supervision process is done frequently including calling CHWs before collecting monthly reports through phone calls and visits. The reporting method is still using paper. Feedback is provided by the coordinator by providing necessary support to CHWs such as informing us whenever medicines and other supplies have been made available at the health facility, keeping important information, and updating information for CHWs. | Not explained in detail, the supervision assessment mechanism is only limited to collecting and coordinating. However, the result is that supervision has succeeded in increasing CHW performance. |
| Kok 2019, [^24^](https://www.zotero.org/google-docs/?iypXtx) Tanzania | Family planning, post-abortion care, cervical cancer | N/A | Level 1:  Monthly progress review meetings include receiving monthly progress reports and the clinic manager or clinic administrative assistant accompanies all monthly awareness raising activities and provides direct feedback to the CBMs.  Level 2:  Monthly progress review meeting and MST head office visits the CBMs in the field once every 6 months and takes a group photo. | The output of this intervention is the motivation of CHWs. Level 2 supervision was more preferred/seen as an external motivation by CHWs. |
| Oluwole 2019, [^62^](https://www.zotero.org/google-docs/?vnV929) Nigeria | Neglected Tropical Diseases (NTDs) | Primary healthcare workers and NTD teams. | Supervision is carried out by visiting the CHW directly. Form reports using paper-based. Many CDDs (Community Drug Distributor) reported that there was no supervision from the FLHF (First Level Health Facilities) during MAM (mass administration of medicines). CDD only communicates with FLHF when reporting data only. Teachers also reported a lack of support from health workers, especially in managing the side effects of using praziquantel. | Supervision is not running effectively. Most of the CDD and teachers stated that there was rarely supervision. According to health workers, FLHF has problems, namely they receive less training, so knowledge about NTDs is minimal, apart from that there is a high workload, and work commitment factors. FLHF also revealed that they had transportation problems so they couldn't carry out routine supervision. Teachers reported that MAM sometimes coincided with school exams, during which teachers were busy preparing for exams and students themselves were also focusing on exam moments. CDD and teachers stated that they needed help from local government, school principals, school management to help them campaign for eradicating worms in schools. |
| Strodel 2019, [^63^](https://www.zotero.org/google-docs/?Gg1xbn) India | 1940-1960s: multiple interventions  1960-1970s: specific targeted interventions (family planning, immunisations)  1990s: not explained | Community, however it is often delegated to local PHC (by health workers). The PHC would provide technical support and hire an additional medical officer to support the new workers | There are no formal documents or processes on how the community should conduct supervision to the CHWs. | Supervision scheme was identified as one of many factors contributing to the failure of the CHW or VHG (Village Health Guides) scheme at that time in India. In the theoretical outline of the scheme, VHGs were meant to be supervised by the community. In practice, however, this task was often delegated to the local PHC, deemphasizing the community-centred goals of the VHG program.  Major barriers to scaling up VHGs Scheme, including insufficient incentives; weak CHW supervision; a lack of community support for the CHW; and poor reception of CHWs into the existing health system. |
| Aridi 2014, [^31^](https://www.zotero.org/google-docs/?JSmt2F) Kenya | MCH, NCD, communicable disease | Government CHWs: supervised by the nearest health facility staff, village health committee (CHC), and CHW supervisors. One CHW supervisor supervises 25 CHWs.  MVP CHWs: supervised by health facilitators who also work as health facility staff and CHC. There is one senior CHW in 6 CHWs. Every 8-20 senior CHWs are supervised by one CHW manager. Above the CHW manager there is one health coordinator. | Government CHW: Health facility staff meet CHW once a month to discuss reports and directly supervise CHW activities. CHC is very helpful for CHWs, especially in dealing with challenges when dealing with village communities. Then in 2010 there was a CHW supervisor. However, their work is limited due to difficulties with transportation funds, so they cannot directly supervise the activities of their CHWs, they also lack skills and do not receive training.    MVP CHW: CHW provides biweekly reports to them. There is a special platform for handling malaria through a mobile phone called the 'Child Count'. This platform uses SMS to record treatment and provide appropriate treatment recommendations. The supervisor will come within 24 hours if a case of malaria is found and one day later if there is a report of toddler diarrhoea. There is a toll-free telephone that can be used by CHW to contact supervisors when experiencing problems with home visits, so it does not require the presence of a supervisor in the field. The CHWs are also supervised by the CHC. In contrast to the CHC, CHWs, the working area of government CHWs, the CHC here did not perform its role properly, in fact there were often debates between the CHC and the CHWs, especially regarding working hours. On the other hand, the CHW positively assessed the work of the health facilitators in supervising them and solving their problems. | Government CHW: Supervision by CHW supervisors does not work due to lack of transportation funds for CHW activity visits.    MVP CHWs: The supervision system is running better, especially with the free telephone system which makes monitoring activities run more effectively. |
| John 2020, [^35^](https://www.zotero.org/google-docs/?1akAWe) India | Mother and Child Health, Nutrition | N/A | The supervisor assists the CHW in convincing reluctant beneficiaries to vaccinate their children, and explains to the CHW the monitoring checklist, format and register. Reporting uses paper manuals, and feedback is delivered directly. The results of interviews with CHWs found that there were fierce supervisors and this made CHWs afraid to ask questions. | The article does not explain the supervisory mechanism, but the results of the in-depth interviews state that there are constraints that the supervisor is fierce and scary, so that the CHWs are afraid to ask questions to the supervisor. The results of the study stated that there was a mixed effect of supervision on CHW performance. Supportive supervisor will improve CHW performance. But it would be counterproductive if the supervisor is grumpy and scary |
| Chipukuma 2020, [^33^](https://www.zotero.org/google-docs/?pDvx0z) Zambia | Malaria Prevention | Supervisor are members of the Church, health centre health workers. The article does not explain how many CHWs are supervised by one supervisor | Supervision is carried out at the facility level but every three months, by the Anglican Church which supports the malaria program. Sometimes the CHW is supervised by a puskesmas health worker during a visit. Supervision is carried out by means of site visits, namely by checking reports and inspection results, as well as holding meetings to discuss the various challenges found. Supervision feedback is carried out to discuss the various challenges found. | The environmental health officer in charge of the CHW did not monitor the CHW on a continuous basis due to limited transportation logistics and a lack of professional staff supervising the CHW, this made the CHW lose motivation to work. The proportion of compliant CHWs showed low performance outcomes and substandard service delivery despite having high knowledge of Malaria. |
| Raven 2020, [^64^](https://www.zotero.org/google-docs/?FCIKNX) Africa Region: Sierra Leone, Liberia, Republic of Congo. | Reproductive, maternal, newborn and child health; integrated community case management of sick children; and infection prevention and control. | Nurse | The supervision process varies from countries.   1. Sierra Leone: Conducts monthly visits to observe CHW work, checks drug supplies and reports and coordinates monthly CHW meetings at the Peripheral Health Unit. Environmental health unit manager makes regular visits to each CHW, attends monthly CHW meetings, provides advice and training to CHWs, distributes drugs and supplies, compiles CHW reports and sends them to the CHW focal person at district level. 2. Liberia: provides field supervision to 10 CHWs working in remote communities, compiles reports from CHWs.   Facility Manager: checks CHW reports and clarifies issues and reports to the district health team.   1. Democratic Republic of Congo:   CHW group leader: organises monthly meetings, reports to the head nurse, who then reports to the District Health Office.  Facility head nurse: makes regular visits to observe CHWs' work and records, provides training when needed, for example when implementing a specific program or when there is an increase in health problems. | CHW supervisors in Sierra Leone and CHSS in Liberia reported the absence of bicycles or transportation allowances as a significant barrier to their work. They often make long journeys, sometimes at their own expense. Meanwhile, the CHW Supervisor in the DRC region said that his supervision was going well. |
| Ashebir 2021, [^89^](https://www.zotero.org/google-docs/?yMrLuD) Ethiopia | Optimization of the Health Extension Program Intervention | PHC health workers | Supervision is only done when the program starts. This is done by giving directions on how to intervene. However, there is currently no support at all. | Supervision mechanisms are not running effectively. Primary health care staff lack supervision because they have many tasks and lack of job satisfaction. As well as the limited number of PHC staff assigned to supervise when compared to the number of WDG CHWs or leaders. The impact on program outputs and achievements is to reduce the motivation of CHWs and weaken their performance in promoting the health of mothers, newborns and children. The absence of incentives given and the lack of training and supervision also caused many CHWs to stop being CHWs.  All respondents stated that the low commitment of PHC health workers was the main obstacle in providing supportive supervision for WDG Leaders. |
| Olaniran 2022, [^66^](https://www.zotero.org/google-docs/?k3NIje) Multi Country study (Bangladesh, India, Kenya, Malawi, Nigeria) | MCH | Professional health workers | Not explained in detail in the article regarding the time, method, report form and supervision feedback. | CHWs who receive positive feedback and public praise from senior colleagues tend to be more motivated, committed, and dedicated to carrying out tasks. Clinical supervision by a healthcare professional is deemed necessary by CHWs who have more specialised roles such as delivering deliveries in India. Moreover, the presence of healthcare professionals during clinical procedures helps to increase the confidence of CHWs and their service recipients as they are assured of receiving prompt assistance from healthcare professionals in cases of clinical complications. The supervisor's visit highlighted the CHW's relationship with the formal health system, especially among community-based CHWs who had no visible link to the formal health system. In effect, it increased the social respect and acceptance of the CHW within the community. |
| Rabbani 2016, [^49^](https://www.zotero.org/google-docs/?zV1Mgm) District Badin of Sindh, Pakistan | Pneumonia and diarrhea | Lady Health Supervisors (LHSs) are an-other CHW of CHWs working in the Lady Health Worker Programme (LHW-P) who are responsible for directly managing 25-30 LHWs (Lady Health Workers). | Supervision processes carried out by LHSs make monthly visits to each LHW to supervise their community case management (CCM) skills during visits to community households. They report to the Assistant Dis-trict Coordinator (ADC) of the LHW-P. Supervisory tools were introduced to supplement the LHW-P management information system (MIS) | Lady health supervisors are motivated by both their role in providing supportive supervision to lady health workers and by the supervisory support received from their coordinators, managers, and fellow health professionals. LHSs are proud of their supervisory skills and are encouraged when their LHWs and fellow LHSs provide good care to communities. It is important to LHSs that they provide meaningful supervision to LHWs by exercising patience and support when teaching. |
| Musoke 2021, [^36^](https://www.zotero.org/google-docs/?SxTGA9) Uganda | Childhood illnesses, maternal and child health, and HIV/AIDS. | CHW coordinators who come from church congregations and stakeholders. | Supervision is carried out with on-site assistance, conducting training. Report forms are site visits and mobile apps. The form of feedback was not explained as to the form of the feedback, but it was explained that the CHW received regular feedback from the supervisor. | Some are not working effectively. As for those that work effectively, such as providing feedback and rewards: Having regular feedback by supervisors and submitting reports are the main motivations for CHW to work. In addition, CHW is also given non-financial rewards such as branded t-shirts and certificates, these rewards motivate CHW to work. As well as the availability of adequate equipment and supplies as the main supply for CHW so that they can continue to respond to community needs.  Ineffective reporting system: Inefficient reporting mechanism among implementing partners including the government. This is because partners use reporting tools and indicators that are different from each other, so this affects the way CHW fills out reports, becomes inefficient and reduces the quality of report content. |
| Dam 2022, [^73^](https://www.zotero.org/google-docs/?m1vGIy) Multiple countries | TB | 89% projects had CHW performance issues addressed by a direct supervisor, while 15% addressed by upper management.  Each supervisor oversaw an average of 33 CHWs, with a higher average in the High-Impact group (15 CHWs per group), compared to those in Medium-Impact group (7 CHWs per group) and Low-Impact Group (13 CHWs per group) | The majority of projects in this survey had CHW performance issues that were dealt with by the direct supervisor without the need for upper management involvement. | There is no significant difference between High-Impact, Medium-Impact, and Low-Impact group for the variables of: average number of supervisor reviews per quarter (6 reviews quarterly in High-Impact group) and average number of supervisor direct feedback per quarter (6 feedbacks quarterly in High-Impact Group).  Supervisors provide direct feedback to CHWs on average 13 times per quarter (3 months), making the conclusion that the regularity of feedback is unlikely to affect project outcomes. However, previous research on the CHW model has also noted that regular supervision can be demotivating as some CHWs perceive their supervision as related to their poor performance. Conversely, more supportive supervision from formal health workers can give CHWs a sense of legitimacy and increase work motivation. |
| Kawakatsu 2022, [^52^](https://www.zotero.org/google-docs/?QzSgFy) Kenya | MCH | Community Health Extension Workers (CHEWs) | Intervention is carried out through >1 supportive supervision CHWs per month. CHEWs are trained as CHW supervisors, by district health officials responsible for community health. CHWs are further trained on key topics related to basic community health. CHWs and local leaders in their catchment communities are responsible for identifying and sharing training outcomes with the community. | CHWs who were supervised once a month or more increased their knowledge by 9.7% and the coverage of home visits was 13% compared to CHWs who were supervised once a month or less. Provision of tracking training is accompanied by supervision which often increases the knowledge of CHWs 17 times higher than those who do not. The combination of monthly refresher training plus loss to follow-up and >1 CHW support monitoring per month is likely to create a greater synergistic effect in increasing and maintaining CHW health knowledge than the other combination (22 times higher than not receiving frequent refresher and monitoring training). |
| Pandya 2022, [^45^](https://www.zotero.org/google-docs/?GPh6ft) Uganda | MCH | Health Officer | The details of supervision (time, method, form of report and feedback) are not discussed in this article. | CHWs in this study often highlight the problem of lack of recognition and respect from health facility staff, which affects their credibility in society. The direct impact on program outcomes is not explained |
| Toney 2022, [^74^](https://www.zotero.org/google-docs/?rIDwgS)Nebraska | Primary Health Care, MCH | CHW was supervised by CHW Supervisor. | Supervision is carried out through site visits by providing feedback through face-to-face meetings with CHW | Most of CHWs said their supervisors were supportive, willing to facilitate training and other professional development. Others think their supervisors are unsupportive, lack communication skills, never give feedback or give advice if CHW has problems in the field. Supervisors believe that having incentives will motivate the CHWs, but in fact some CHWs say program incentives stress them out. |
| Aftab 2018, [^30^](https://www.zotero.org/google-docs/?0KHf0n) Pakistan | MCH | Each supervisor supervises 25-30 CHWs. called Lady Health Supervisor (LHS) | Routine supervision system:  Supervision is carried out twice a month by observing the performance of CHWs and providing verbal feedback. CHWs make a report to the supervisor at the monthly meeting. Then the supervisor compiles the data and sends it to the programmers at the regional level.  Intervention supervision system:  Supervisors are being trained again, material refreshing, mentorship and supervision training and how to write feedback. They were given cell phones and communication money. The CHW sends an SMS to the supervisor if they find a case within 24 hours. Then the supervisor immediately came for supervision. Supervisor reviews clinical findings and management plan developed by CHWs. SMS is also used to identify cases for field skills assessment. There is a feedback sheet for direct observation of CHWs performance. | Supervision performance shows a sharp increase, especially in how to demonstrate correct clinical methods to CHWs and provide feedback. The intervention supervision mechanism has the potential to improve the performance of CHWs through strengthening communication, monitoring and accountability mechanisms. However, it must be accompanied by political will, training, logistics, and supply of commodities. |
| Roy 2022, [^67^](https://www.zotero.org/google-docs/?NZGfOW) Bangladesh | CHW- FWA: communities and clinics  CHW- HA: the Expanded Programme on Immunization (EPI) and disease surveillance and provide other primary health care services. | FWAs directly report to FP inspectors and HAs directly report to assistant health inspectors/health inspectors. | Both FWAs and HAs are expected to check in at least monthly with their supervisors. Regular offline team meetings and through digital communication (whatsapp, text, etc.), Zoom-meeting/G-meet, but also prioritising flexibility. CHW stated that they prefer to supervise directly face to face, but the supervisor suggested that CHW could meet face to face if there were urgent meetings or problems that had to be resolved in person (offline). There are reports and feedback but the mechanism is not explained in the article. | The supervision mechanism is running effectively. Most CHWs reported having had supportive supervision during the pandemic. Supervision is carried out online, but CHW training is still carried out offline. If there is an urgent and important matter, the CHW may meet with the supervisor offline. |
| O'Donovan 2022, [^42^](https://www.zotero.org/google-docs/?520sB8) Uganda | Child and maternal | Seven of the 14 CHWs considered staff of NGO A to be their supervisor; five stated it was a fellow CHW;    Two stated it was a member of staff at the health facility, or a mixture of NGO staff and fellow CHWs. | The majority of CHWs (12 CHW) receive supervision every 3 months, but (1 CHW) there are those who do every month and (1 CHW) the time of supervision is not certain. About the method of supervision, the majority of CHWs (10 CHW) receive supervision by means of group supervision, (2 CHW) in-person one-to-one supervision, (1 CHW) one through mobile phone. Meanwhile about the duration of supervision, 12 of the 14 CHWs stated that supervision sessions lasted longer than 60 min, whereas the remaining two stated that they lasted between 15-30 min | CHWs and supervisors stated that supervision activities were ineffective due to limited funds for mobility in carrying out or participating in supervision. Also there is the need for detailed explanation of duties and training for supervisors. CHWs feel appreciated and meaningful when receiving supervision, thereby encouraging CHW motivation  From a stakeholder perspective, there are three qualities that are necessary to become a "good supervisor", namely:   1. Technical knowledge and expertise: Have good knowledge in terms of technical and clinical skills so that it is easier to give feedback to CHWs. 2. Interpersonal skills: These skills include active listening, trustworthiness, confidentiality, empathy, being non-judgemental and approachable 3. Cultural awareness: Having the ability to adapt to local culture, for example language, customs, etc. |
| Nakibaala 2022, [^70^](https://www.zotero.org/google-docs/?6PmZg4) Uganda | N/A | 1. Community Health Supervisor (CHS): Full-time employee of the institution. 2. In selected areas: some CHWs performed as peer supervisors. | Each CHS is looking for 5-10 groups. 1 group consists of 8-12 CHWs that were choosing their own peers to become a role model (peer supervision). Supervision by CHS carried out by monthly meetings, coach the peer supervisors, review performance and quality, conduct group field visits/meetings, re-stock CHWs, and conduct refresher training as needed. Meanwhile Peer supervisor responsibilities are motivate the CHWs to achieve their targets, review CHW performance, CHW stock check, door to door activities/ movement with CHWS | The number of CHWs achieving KPIs is statistically significantly higher in CHWs who had peer supervision. Attrition (leaving the institution) is lower in CHWs who had peer supervision. Completeness of high-impact medical utilities are higher in CHWs who had peer supervision. Very good satisfaction from CHWs. Less costly |
